# Air pollution and children’s health—a review of adverse effects associated with prenatal exposure from fine to ultrafine particulate matter

**DOI:** 10.1186/s12199-021-00995-5

**Published:** 2021-07-12

**Authors:** Natalie M. Johnson, Aline Rodrigues Hoffmann, Jonathan C. Behlen, Carmen Lau, Drew Pendleton, Navada Harvey, Ross Shore, Yixin Li, Jingshu Chen, Yanan Tian, Renyi Zhang

**Affiliations:** 1 Department of Environmental and Occupational Health, Texas A&M University, College Station, TX 77843 USA; 2 Department of Veterinary Pathobiology, Texas A&M University, College Station, TX 77843 USA; 3 Department of Chemistry, Texas A&M University, College Station, TX 77843 USA; 4 Department of Veterinary Physiology and Pharmacology, Texas A&M University, College Station, TX 77843 USA

**Keywords:** Air pollution, Particulate matter, PM_2.5_, Ultrafine particles, Prenatal exposure, Children’s environmental health, Health effects

## Abstract

**Background:**

Particulate matter (PM), a major component of ambient air pollution, accounts for a substantial burden of diseases and fatality worldwide. Maternal exposure to PM during pregnancy is particularly harmful to children’s health since this is a phase of rapid human growth and development.

**Method:**

In this review, we synthesize the scientific evidence on adverse health outcomes in children following prenatal exposure to the smallest toxic components, fine (PM_2.5_) and ultrafine (PM_0.1_) PM. We highlight the established and emerging findings from epidemiologic studies and experimental models.

**Results:**

Maternal exposure to fine and ultrafine PM directly and indirectly yields numerous adverse birth outcomes and impacts on children’s respiratory systems, immune status, brain development, and cardiometabolic health. The biological mechanisms underlying adverse effects include direct placental translocation of ultrafine particles, placental and systemic maternal oxidative stress and inflammation elicited by both fine and ultrafine PM, epigenetic changes, and potential endocrine effects that influence long-term health.

**Conclusion:**

Policies to reduce maternal exposure and health consequences in children should be a high priority. PM_2.5_ levels are regulated, yet it is recognized that minority and low socioeconomic status groups experience disproportionate exposures. Moreover, PM_0.1_ levels are not routinely measured or currently regulated. Consequently, preventive strategies that inform neighborhood/regional planning and clinical/nutritional recommendations are needed to mitigate maternal exposure and ultimately protect children’s health.

**Supplementary Information:**

The online version contains supplementary material available at 10.1186/s12199-021-00995-5.

## Background

**Fig. 1:**
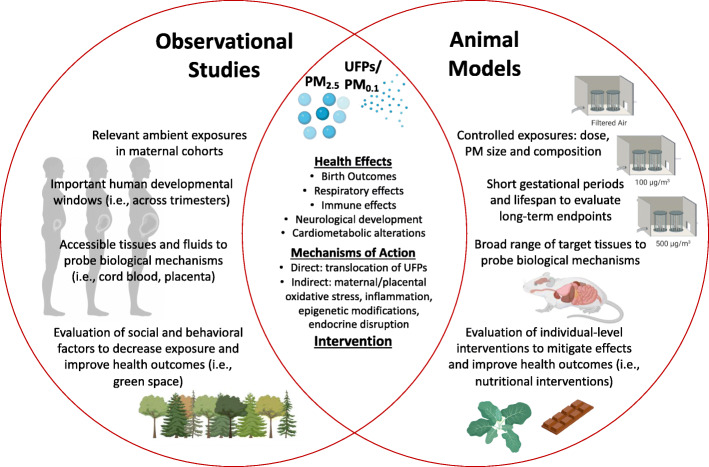
Evidence gleaned from human observational studies and animal models is essential in characterizing adverse health outcomes and deciphering the mechanisms of action to support preventive intervention testing. Selected strengths of these two methodological approaches are highlighted in the Venn diagram. In this review, we synthesize the scientific evidence on adverse health outcomes in children following prenatal exposure to fine particulate matter (PM_2.5_) and ultrafine particles (UFPs, PM_0.1_). Additionally, we summarize the evidence on underlying mechanisms of action. Created with BioRender.com

Human exposure to ambient air pollution is a pervasive public health issue based on the substantial cause of disease and death worldwide [1]. Suspended aerosols known as particulate matter (PM) are a predominant toxic component of ambient air pollution emitted by a variety of sources, including vehicular traffic, coal-burning power plants, waste burning, and other industrial activities. Particulate matter (PM) is classified by size as either “coarse” (PM_10_) with an aerodynamic diameter less than 10 μm, “fine” (PM_2.5_) with a diameter less than 2.5 μm, or “ultrafine” (PM_0.1_) with a diameter less than 0.1 μm. The fine and ultrafine fractions can penetrate deeper in the airways in comparison to coarse particles, leading to numerous adverse health effects. A wealth of evidence highlights maternal exposure to pollutants during pregnancy represents a window of susceptibility for fetal development and children’s long-term health. For instance, it is well-established that early life tobacco smoke exposure increases the risk of respiratory infection and asthma in infancy and childhood [2, 3]. Analogous to tobacco smoke, developmental exposure to PM_2.5_ has been intensively investigated in human epidemiological studies. Outcomes at birth and early respiratory effects are extensively reviewed elsewhere [4–6]. In this review, we will provide an overview of these findings, including emerging data related to ultrafine PM exposure. Additionally, data on neurological effects and cardiometabolic disease risk continue to emerge, and key findings are highlighted [7–9]. Laboratory-based inhalation toxicology studies using in vivo models are also instrumental in establishing causality between prenatal fine and ultrafine PM exposure and adverse outcomes observed in human populations. A main objective of this review was to comprehensively examine experimental models evaluating early life exposure to PM_2.5_ and/or PM_0.1_ and effects on offspring. Additionally, we summarize the evidence on underlying mechanisms of action gleaned from human and nonhuman studies. Last, we briefly summarize preventive intervention strategies related to mitigating maternal exposure, but detailed behavioral, neighborhood, and nutritional interventions is beyond the scope of this review. Overall, we highlight the evidence gleaned from human observational studies and animal models is essential in characterizing adverse health outcomes and deciphering the mechanisms of action to support preventive strategies to ultimately protect children’s health (Fig. 1).

## Study selection

Five literature searches were performed using PubMed (details in Supplemental file). The search strategy combined key sets of words using AND/OR. In the first search, terms included “prenatal” AND “particulate matter” OR “ultrafine.” Two independent reviewers (NMJ and an additional content author) scanned titles and abstracts to determine eligibility and to further categorize studies into human or nonhuman evidence. Subsequently, studies were categorized by endpoints, such as birth outcomes, respiratory/immune, brain, and cardiometabolic. We included original studies that evaluated prenatal exposure to ambient fine or ultrafine PM. We excluded studies if (1) the article did not report ambient PM_2.5_ or PM_0.1_ exposure; (2) the article contained no original data related to human on nonhuman (rodent) health effects in each pre-defined endpoint category; or (3) other reason, with an explanation required. All duplicate articles were removed. Two independent reviewers reviewed the full-text articles for inclusion and excluded studies that were duplicates, nonrodent, or not related to their primary endpoint. Four additional searches were carried out the same as above using 1: “particulate matter” AND “pregnancy” AND “oxidative stress”; 2: “particulate matter” AND “pregnancy” and “inflammation”; 3: “particulate matter” AND “pregnancy” AND “epigenetic”; and 4: “particulate matter” AND “pregnancy” AND “endocrine.”

## Human evidence from epidemiologic studies

### Adverse birth outcomes

There is a strong body of human evidence from epidemiological studies associating PM, in particular PM_2.5_, with adverse birth outcomes. Systematic reviews with meta-analyses [10–13], summarized in Table 1, demonstrate positive, and often significant, associations between PM_2.5_ exposure across the entire pregnancy and increased risk of preterm birth and infant low birth weight. Effect estimates for every 10 μg/m^3^ increase in PM_2.5_ exhibit similar ranges across analyses for reductions in birth weight from −15.9 to −23.4 g. Studies have also interrogated the impact of exposure during specific trimesters to identify critical windows of susceptibility. Overall, the results are mixed; however, a common theme of increased impact appears for exposure later in pregnancy. DeFranco et al. [14] also observed the greatest risk for preterm birth (19% increased risk) in the third trimester. Percy et al. [15] demonstrated increasing exposure to PM_2.5_ during the third trimester, in particular between 30 and 35 weeks of gestation, was associated with an increased risk for small for gestational age babies. In an observational natural experiment, infants whose 8th month of gestation occurred during the 2008 Beijing Olympics, when air pollution levels drastically deceased, were born 23 g heavier on average compared to infants whose 8th month occurred over the same dates the year prior (2007) or after (2009) [16]. It is plausible exposure later in pregnancy, during periods of rapid fetal weight gain, has a larger impact on infant birth weight. Likewise, stressors closer to delivery could affect preterm birth. However, for other adverse outcomes, such as cognitive effects, earlier exposure during neurogenesis may be considered the critical window. Continued research incorporating smaller windows, such as weeks, may help further illuminate critical windows of susceptibility.

**Table 1:** Summary of meta-analyses results related to preterm birth or infant low birth weight and PM_2.5_ exposure across pregnancy

Reference	Odds ratio for preterm birth (95% CI)	Odds ratio for low birth weight (95% CI)	Effect estimate (g) for every10 μg/m^3^ increase in PM_2.5_
Stieb et al. [10]	1.05 (0.98, 1.13) *n = 4*	1.05 (0.99, 1.12) *n = 6*	−23.4 (−45.5, −1.4) *n = 7*
Zhu et al. [11]	1.10 (1.03, 1.18) *n = 8*	1.05 (1.02, 1.07) *n = 6*	−14.6 (−19.3, −9.9) *n = 12*
Lamichhane et al. [12]	1.13 (0.98, 1.28) *n = 5*	NA	−22.2 (−37.9, −6.4) *n = 7*
Sun et al. [13]	NA	1.09 (1.03, 1.15) *n = 19*	−15.9 (−26.8, −5.0) *n = 17*

The risk of stillbirth has also been investigated in association with prenatal exposure to PM_2.5_. A meta-analysis including 13 studies cited exposure to ambient air pollution increases the risk of stillbirth; however, PM_2.5_ alone was neither found to be statistically significant [17], nor was it significant in the meta-analysis performed by Zhu et al. [11]. Similarly, in an Ohio cohort, “high” PM_2.5_ exposure, defined as greater than or equal to the mean PM_2.5_ level during the study period (13.3 μg/m^3^) plus the IQR for the specific time period measured for each birth) was not associated with increase in stillbirth risk through pregnancy, first or second trimester; however, high PM_2.5_ exposure during the third trimester was associated with 42% increased stillbirth risk [18]. Further epidemiological and mechanistic studies are needed to validate the causal linkage between PM_2.5_ exposure and stillbirth risk.

### Respiratory effects and impact on the immune system

Prenatal exposure to PM_2.5_ impacts lung development and respiratory health in a variety of ways that may persist throughout childhood [5]. Developmental PM_2.5_ exposure can lead to disturbed alveolarization, impaired lung function, and pulmonary immune differentiation, which may influence acute and chronic health outcomes. In a meta-analysis of multiple European birth cohorts, MacIntyre et al. [19] concluded there was consistent evidence for an association between air pollution and pneumonia in early childhood. The link between prenatal PM_2.5_ exposure and the development of asthma has also become increasingly recognized as epidemiologic studies have reported positive associations [6, 20]. Hehua et al. [6] reviewed 18 studies and found that children prenatally exposed to multiple air pollutants had increased risk of wheeze and asthma during childhood. However, only a weak association was identified in the five existing studies that evaluated prenatal PM_2.5_ exposure. These findings highlight a research gap and the need for future studies to examine PM_2.5_ exposure and contribution to asthma etiology, as well as mechanistic studies to tease apart the complex gene-environment interactions involved in asthma development.

PM_2.5_ also affects the immune system, although only a handful of human studies relevant to early life exposure are published to date [21, 22]. Herr et al. [23] observed that prenatal exposure to PM_2.5_ shifted lymphocyte distributions representative of the neonatal adaptive immune response in umbilical cord blood. Exposure during early gestation resulted in increased T lymphocytes, decreased B lymphocytes, and natural killer cells, whereas late gestation exposure was associated with an opposite immune phenotype with decreased T lymphocytes, increased B lymphocytes, and natural killer cells. Since infants are at risk for respiratory infections, the impact of PM_2.5_ on neonatal immunity may significantly influence morbidity risk [24].

### Effects on neurological development

The importance of cognitive function and increasing prevalence of neurodevelopment disorders, including developmental delay, attention deficit hyperactivity disorder (ADHD), and autism spectrum disorders (ASD) has spurred a large amount of research examining early life exposure to ambient air pollution [7, 25, 26]. In general, epidemiological studies have investigated the impact of prenatal exposure to PM_2.5_ on structural alterations, cognitive function, and risk of clinically defined disorders. In a study examining prenatal PM_2.5_ exposure, children presented with thin cortex in many regions of the brain and an impaired inhibitory control. Impaired inhibitory control is related to other mental health problems, including addictive behavior and ADHD [27]. Mortamais et al. [28] estimated prenatal PM_2.5_ levels at maternal residential addresses during the 3rd trimester of pregnancy and observed an increase of 7 μg/m^3^ was significantly associated with a decreased corpus collosum (the bridge connecting the two hemispheres) body volume. This decreased volume was linked with higher hyperactivity scores, indicative of behavioral issues. Prenatal PM_2.5_ exposure has also been correlated with reduced fundamental cognitive abilities, including working memory and conflict attentional network [29]. In a systematic review of multiple airborne pollutants and ASD risk, Lam et al. [26] concluded the strongest evidence was between prenatal exposure to PM_2.5_. However, the small number of studies (n = 3) in the meta-analysis and unexplained heterogeneity signified that the effect could be larger or smaller than those studies estimate, supporting the need for additional research on PM_2.5_ exposure and ASD risk. Overall, the mounting human evidence suggests developmental PM_2.5_ exposure effects neurobehavioral function and contributes to cognitive impairment. Advancing air pollution policy for the protection of children’s health and promotion of healthy brains, similar to prevention of lead poisoning and mitigation of cognitive deficits, is warranted on the basis of this burgeoning epidemiological evidence [9].

### Metabolic alterations

In the USA, the prevalence of childhood obesity has nearly doubled since 2000 [30]. Over 18 million children are obese, 19% of the population in 2015-2016, in comparison to less than 10% of the population in 1999-2000. Parallel to this increase in obesity is an elevated prevalence of type 2 diabetes [31]. While the etiology of type 2 diabetes is multi-factorial, obesity is a primary risk factor as this state disrupts insulin homeostasis leading to abnormal blood glucose levels [32]. Studies looking at early life environmental influences on the development of metabolic disorders have emerged in the face of the current obesity epidemic [33]. A handful of studies have evaluated how developmental air pollution exposure impacts offspring body mass and metabolic disease risk. Alderete et al. [34] demonstrated that prenatal residential traffic-related air pollution exposure was associated with higher adipokines, leptin, and adiponectin levels in umbilical cord blood. Increased leptin levels correlated with significant weight gain in female infants, which investigators reasoned could increase future obesity risk. In subsequent work, researchers observed higher NO_2_ and PM_2.5_ levels were correlated with altered β cell function and insulin sensitivity and was associated with a higher body mass index (BMI) at 18 years of age [35]. In another study, infants exposed to higher traffic-related air pollution in early life were more likely to develop the “thrifty phenotype,” where infants initially born with low birth weight were more prone to rapid weight gain in the first 6 months of life [36]. Specific to prenatal PM_2.5_ exposure in the 3rd trimester, effect estimates were in the same direction, but smaller and imprecise. The same investigators followed a cohort of 1400 children that lived close to a highway during delivery. Children were found to have increased adipose tissue accumulation during early and mid-childhood [37]. Paradoxically, PM_2.5_ exposure improved cardiometabolic markers. In a follow-up study, closer residential proximity to freeways was associated with a higher BMI in early childhood, but showed no evidence of a persistent effect [38]. In a German cohort, Thiering et al. [39] showed insulin resistance was greater in children at 10 years of age who were exposed early in life to traffic-related air pollution. In a follow-up study, landscape “greenness” attenuated the effect, which could be attributable to lower pollution exposure [40]. Moody et al. [41] observed prenatal and perinatal PM_2.5_ exposure was associated with changes in HbA1c levels in early childhood. This marker is indicative of glucose dysregulation, providing evidence that early life exposure may influence diabetes risk. Another study demonstrated that third trimester PM_2.5_ exposure led to increased risk of offspring high blood pressure, which could further lead to cardiometabolic dysfunctions [42]. Complicating research on hypertensive disorders, gestational hypertension is associated with higher offspring blood pressure; several studies show maternal PM_2.5_ exposure may increase the risk of gestational hypertension or preeclampsia, a risky pregnancy complication characterized by high blood pressure [43–45]. In summary, additional research is needed on how PM_2.5_ exposure during pregnancy leads to metabolic alterations in children later in life.

### Summary of human evidence

Based on the numerous epidemiological studies summarized above, it is clear that prenatal exposure to fine PM is associated with numerous adverse effects in children, including acute birth outcomes and chronic respiratory effects, with a growing body of literature indicating cognitive and metabolic dysfunction. Although not currently regulated through air quality standards, ultrafine particles (UFPs, PM_0.1_) are postulated to exert enhanced toxicities due to their larger surface area/mass ratio, enhanced oxidative capacity and ability to translocate into systemic circulation [46]. In general, there is a lack of human evidence on the specific effects from prenatal exposure to UFPs, in part due to the lack of monitoring and models to estimate UFP exposure [47]. In the first large-scale epidemiologic study, Lavigne et al. [48] demonstrated in addition to PM_2.5_ and nitrate exposure, prenatal UFP exposure was independently associated with childhood asthma incidence. Wright et al. [49] also observed prenatal UFP exposure was associated with asthma development in children in the Northeastern USA, independent of NO_2_ and temperature. These emerging findings emphasize the need to fill the current gap in the literature interrogating the relationship between prenatal UFP exposure and adverse health outcomes in offspring, particularly immune, neurological, and cardiometabolic endpoints. Further data will help verify the independent risk of UFP exposure on developmental endpoints, as well as inform the effects from multi-pollutant models. In the scarcity of human evidence, a variety of recent studies in animal models have investigated the specific effects of UFP exposure. Described in more detail in the next section, phenotypic and mechanistic data gleaned from animal models help support improved knowledge and encourage further regulation of air pollution exposure during this critical window of development.

## Nonhuman evidence from experimental models

### Experimental approaches

Animal models provide controlled exposure conditions across defined developmental periods to aid in determining dose-response gradients and biological mechanisms of action (Fig. 1). Rodent models, both rats and mice, have been extensively employed to research the effects of prenatal fine and ultrafine PM exposure on offspring. Short gestational windows (approximately 19-21 days for mice and 21-23 days for rats) and rapid offspring development to maturity allow investigators to conduct experiments relatively quick in comparison to human cohort studies that require years before developmental endpoints can be assessed. The broad term “early life exposure” is commonly used in the human and nonhuman literature to indicate prenatal, neonatal, and perhaps childhood exposure. The following section summarizing the nonhuman evidence from rodent exposure models defines the prenatal window as exposure occurring in utero, i.e., during gestation. Sometimes, investigators design experimental studies to encompass the perinatal period and employ exposures after birth. This is based on developmental endpoints, since rodent exposures in early infancy (i.e., the neonatal period) mimic human 3rd trimester exposures for several organs, including the lung [50] and brain [51]. In reality, human exposure can occur before, during, and after pregnancy, and animal models allow researchers to control the precise timing of exposures, which helps define critical windows of susceptibility.

In addition to controlling timing of exposure in models, researchers can regulate the size and dose of PM. Methods for generating and characterizing PM in inhalation toxicology models are reviewed in detail by Chen and Lippmann [52]. In general, models of pre- and perinatal exposures have employed various agents (i.e., components of PM) and particle sizes (fine and/or ultrafine fractions) using different routes, most frequently inhalation not only via whole-body or nose-only chambers but also intranasal and intratracheal instillation in some cases. Stress induced from confinement within nose-only chambers and the anesthesia required for particle instillation are limitations of particular importance when working with pregnant dams. A few investigators have utilized ambient air exposures through direct exposure to traffic or polluted urban air [53, 54]. Direct exposure to traffic and ambient urban air can vary temporally, thus, may limit reproducibility. However, a strength is environmentally relevant atmospheric concentrations. For instance, exposures have averaged around 16.8 ± 8.3 (SD) μg/m^3^ in São Paulo [53] to 73.5 ± 61.3 (SD) μg/m^3^ in Beijing [54].

Several research teams have utilized concentrated ambient particulate matter systems (CAPs) to deliver fine and ultrafine PM mixtures to pregnant mice and neonates [52]. One example is the Harvard University Concentrated Ambient Particle System (HUCAPS) fitted with a size-selective inlet for ultrafine particles (< 100 nm) applied in numerous early life exposure models [55–60]. The HUCAPS system concentrates ultrafine particles approximately ten times that of ambient air concentrations. The gas-phase components of the ambient aerosol are present but are not concentrated by the system. PM levels generated have averaged around 67.9 μg/m^3^ (1.82 × 105 particles/m^3^) to 96.4 μg/m^3^ (2.02 × 10^5^ particles/m^3^), reflecting particle numbers and concentrations reported in U.S. cities [55]. Additionally, studies have been carried out using comparable systems, such as the New York University Versatile Ambient Particle Concentrator Exposure System (VACES) [61–63] and the Ohio State University OASIS-1 aerosol concentration system [64–66]. The VACES system concentrates ambient PM at an equivalent factor to the HUCAPS system, with a slightly larger particle size distribution within the fine and ultrafine range. Using the VACES system, Klocke et al. [61] generated an average concentration of 92.69 ± 19.16 (SD) μg/m^3^, and Church et al. [63] produced average levels of 135.8 ± 13.17 μg/m^3^, representing 11 times ambient air concentrations. Similar average levels were recorded for concentrated PM_2.5_ from the Columbus, OH region [64–66]. Overall, these systems have generated human relevant exposures for experimental testing.

In addition to concentrated ambient particulate matter systems, aerosolizing PM from a defined source represents a complementary approach refined in its ability to generate consistent daily PM concentrations from distinct sources. Zhang and colleagues generated an ultrafine PM mixture from a multicomponent aerosol mixture representative of PM chemical composition under typical polluted urban environments [67] using an atomizer and diluted solution consisting of organics, sulfates, nitrates, ammonium, chloride, and diesel exhaust PM [68]. Particle size ranged from 20 to 220 nm, with a peak diameter of 50 nm. Using this system, Rychlik et al. [68] reported an average mass concentration of 101.94 μg/m^3^, corresponding to a 24-h daily mean dose of 25 μg/m^3^. Notably, while there is no current regulatory standard for ultrafine PM, this level is under the U.S. EPA national ambient air quality standard of 35 μg/m^3^ for PM_2.5_ and similar to the WHO recommended guideline of 25 μg/m^3^ for 24-h average exposure. Using the same system, Wu et al. [69] investigated the impact of ultrafine ammonium sulfate particles (peak diameter of 10-20 nm) on offspring development. This exposure replicated several key PM properties observed during polluted haze events in Asia, including chemical composition, size, hygroscopicity, and acidity [70]. Cormier and colleagues have also investigated the specific toxic effects of combustion-generated environmentally persistent-free radicals (EPFR) [71] adsorbed to ultrafine particles in several exposure models [72–76]. EPFRs denote integrated pollutant-particle systems consisting of phenoxyl- and semiquinone-type radicals formed and stabilized by transition metal oxide–containing particles. These represent exposures identified in airborne PM at abandoned hazardous waste facilities that fall under the Comprehensive Environmental Response, Compensation, and Liability Act of 1980, termed Superfund sites [77]. These in vivo inhalation models mimic environmentally relevant doses (200 μg/m^3^), providing an alveolar deposition dose to neonates equivalent to deposition in human infants [73]. Other research groups have applied similar approaches to probe the effects of exposure to fossil fuel combustibles, such as aerosolized residual oil fly ash [78] and vehicular-derived PM [79–81]. In several models, Morgan and colleagues have applied re-aerosolized nano-scale PM (< 200 nm) collected from a heavy traffic site nearby the Los Angeles I-110 Freeway using a high-volume ultrafine particle sampler [79, 80]. Researchers have also re-aerosolized diesel exhaust particles (DEPs) in numerous in vivo inhalation models using engine-produced particles [82], including National Institute of Standards and Technology (NIST) standard reference materials [81].

Alternatively, other groups have applied DEPs in prenatal exposure models via intranasal application [83, 84], oropharyngeal administration [85], and intratracheal instillation [86, 87]. Outcomes from instillation and inhalation exposures can result in differing pathological consequences [88]; however, in the case of in utero exposures, translocation of the particles into systemic circulation may be of greater consequence to fetal development. Investigators have taken care to apply relevant exposure concentrations using these techniques. For instance, Chen et al. [87] applied 20 μg of a DEP suspension, representing an average daily dose of 8.6 μg/mouse, approximately equating to an inhalational exposure level of 160 μg/m^3^ PM_2.5_. Instillation has also been used in general for PM_2.5_ [89, 90] and PM_0.1_ [91, 92] dosing using particles collected in urban environments. Oral gavage [93] and intraperitoneal (i.p.) injection of particles [94] are much less commonly used; however, authors cite technical advantages in comparison to intratracheal instillation. While systemic administration of particles does not represent a physiology route of exposure [95], translocation into systemic and placenta circulation may serve as a proxy to investigate fetal/offspring effects. Last, traffic-related air pollution has been extensively studied in animal models via direct exposure to freshly generated diesel exhaust (DE) [96]. Here, rodents are exposed to both particulate and gaseous components. In several prenatal mouse exposure models, DE concentrations have ranged from 90 to 300 μg/m^3^ PM_2.5_ [97–100]. Overall, the many inhalation toxicology methods for generating controlled PM exposures has spurred substantial research in rodent models demonstrating effects on offspring respiratory, immune, neurological, and cardiometabolic development (described in detail in this section). These findings bolster the results gleaned from human epidemiological studies and provide a deeper understanding of the underlying biological mechanisms.

### Developmental effects

Studies conducted in rodent models have demonstrated varying degrees of adverse birth outcomes following developmental exposure to PM (Table 2). Some models report no effects on outcomes, such as abortion, stillbirth, intrauterine growth restriction, or impact on birth weight. Limitations are associated with assessing some early outcomes, such as stillbirth, as well as differences in models, including species, strain, timing, and type of exposure. There is broad consensus on initial pollutant-induced growth restriction, with more variation in the long-term effects of altered offspring growth trajectories that is driven by differences in exposure models.

**Table 2:** Summary of developmental effects from in vivo models (7 studies)

Reference	Animal model	PM source	Dose	Route	Duration	Offspring effects
Tsukue et al. [101]	C57BL/6J mice	Diesel exhaust	0.3, 1.0, or 3.0 mg DEP/m^3^	Inhalation	4 months pre-mating exposure (12 h/day 7 days/week)	Decreased BW in both sexes; AGD lengths shorter; organ weights less, and vaginal orifices of young females opened significantly earlier (exposed to 0.3 and 1.0 mg DEP/m^3^)
Hougaard et al. [81]	C57BL/6J mice	Diesel exhaust particles	~19 mg/m^3^	Inhalation	GD9–GD19 (1 h/day)	Decreased weight gain during lactation; cognitive function and biomarkers were generally similar across offspring
Gorr et al. [64]	FVB mice	PM_2.5_	51.69 μg/m^3^	Inhalation	Gestation/nursing (6 h/day, 7 days/week in utero until weaning at 3 weeks of age)	Reduced birth weight; at adulthood: reduced left ventricular fractional shortening; reduced ejection fraction; increased end-systolic volume; and reduced dP/dt maximum and minimum; alerted cardiomyocytes profiles; increased collagen deposition
Liu et al. [89]	Sprague Dawley rats	PM_2.5_	15 mg/kg	Intratracheal	GD10 and GD18	Increased absorbed blastocysts; lower maternal weight gain and fetal weight; significant increase of blood mono- nuclear cells, platelets, and IL-6; placenta pathological examination demonstrated thrombus and chorioamnionitis
Chen et al. [66]	C57BL/6J mice	Diesel exhaust particles	8.6 μg/mouse (~160 μg/m^3^)	Intratracheal	3 times/week (M/W/F) beginning at 5 weeks and ending at weaning. (as mating started at 12 weeks, there was approximately a 7-week preconceptional instillation)	No impact to birth weight, however exposure significantly decreased offspring body weight from postnatal week 2 until the end of observation; decreased food intake but no alteration in brown adipose tissue
Chen et al. [87]	C57BL/6J mice	Concentrated ambient particles, PM_2.5_	88.66 μg/m^3^	Inhalation	Preconception, pregnancy, and lactation (6 h/day, 5 days/week; no exposure took place during weekends or the day of birth)	Significantly decreased offspring birth weight, but increased body weight of adult male; adult males had increased food intake, but were sensitive to exogenous leptin
Wu et al. [69]	Sprague Dawley rats	Ultrafine PM, ammonium sulfate	100 to 200 μg/m^3^	Inhalation	Throughout gestation (until GD0-18)	Impacts to prenatal and postnatal organogenesis in offspring; increased stillbirths; reduced gestation length and birth weight; increased concentrations of glucose and free fatty acids in plasma; enhanced lipid accumulation in the liver; decreased endothelium-dependent relaxation of aorta

*BW* birth weight, *AGD* anogenital distance, *GD* gestation day

In a rat model employing 18 days of continuous gestational exposure to ammonium sulfate particles, representative of several key PM_0.1_ properties observed during polluted haze events in Asia, Wu et al. [69] observed a significantly decreased gestational length, percent of offspring alive at birth, and average birth weight in the pollutant-exposed group. After weaning, offspring body weight remained markedly lower, coinciding with a significant reduction in plasma triacylglycerol concentrations. However, at the conclusion of the study (at postnatal day 105), there were no differences in body weight due to prenatal exposure in groups consuming a low- or high-fat diet. Liu et al. [89] exposed pregnant rats to PM_2.5_ on gestation days 10 and 18. Fetal weights were significantly decreased in the PM-exposed group. Moreover, investigators observed increased absorbed blastocysts and abnormal placental pathology following gestational PM_2.5_ exposure. Gorr et al. [64] also observed significantly reduced birth weights in FVB mice exposed to concentrated ambient PM_2.5_ throughout gestation until weaning at three weeks of age. Offspring body weights were slightly higher at 3 months of age in exposed offspring versus control. Absolute heart weights were also increased in the PM_2.5_ group, which correlated with significant cardiovascular dysfunction at adulthood. Manners et al. [84] observed maternal exposure to diesel exhaust PM_2.5_ in a mouse model employing C57Bl/6 mice resulted in offspring with significantly reduced body weights at 4 weeks of age. Other studies have confirmed early reductions in offspring birth weight following prenatal diesel exhaust particle exposure, which were sustained throughout lactation [81]. Chen et al. [87] did not observe an impact on birth weight in offspring of C57Bl/6 mice exposed to diesel exhaust PM_2.5_ during gestation; however, offspring body weight was found to be decreased from postnatal week two until the end of observation (20-22 weeks of age). Reduced weight gain in the PM_2.5_-exposed mice correlated with decreased food intake. Interestingly, reduced weight gain corresponded with increased epididymal adipose tissue mass. In contrast, in a second exposure group, where control mice were fostered by PM_2.5_-exposed dams, offspring body weight increased during lactation and into adulthood (without marked food intake increases). This type of study underscores how differential programming may depend upon timing of exposure.

In a separate study, Chen et al. [66] exposed C57Bl/6 mice to filtered air or concentrated ambient PM_2.5_ during preconception, pregnancy, and lactation or in a second exposure scheme during pregnancy and lactation only. Maternal PM_2.5_ exposure, including the 7-week preconception exposure, resulted in significantly decreased birth weight. Offspring exhibiting lower birth weight had a marked “catch up” growth during the lactation period, beginning after 1 week of age, making them significantly heavier than controls at time of weaning. This increase was maintained in males over the entire observation period (until 18 weeks) but only until 7 weeks of age for female offspring before converging. These data mirror the observed trend in the study by Gorr et al. [64], as well as epidemiological data supporting the “thrifty phenotype” hypothesis [36]. Chen et al. [66] also saw similar trends in the group exposed to PM_2.5_ during pregnancy and lactation only, yet the differences were smaller and failed to reach statistical significance. Contrary to results from Chen et al. [66], diesel exhaust PM_2.5_ preconception exposure at 1.0 or 3.0 DEP/m^3^ led to significantly lower offspring body weights at 8 weeks of age in C57Bl/6 mice [101]. Despite these differences, preconception may represent a window of vulnerability, which is often not included in human observational studies.

### Respiratory and immune system effects

Rodent models employing prenatal fine and ultrafine PM exposure provide substantial evidence on offspring lung dysfunction and increased asthma susceptibility (Table 3). Features of asthma, or allergic airway disease, can be characterized by measuring airway inflammation and hyperresponsiveness following an allergen exposure [108]. Frequently, mouse models employ sensitization and challenge using the experimental allergen ovalbumin (OVA) or a human relevant allergen, house dust mite. Several models employing various exposure regimens and allergen challenges confirm prenatal PM_2.5_ and PM_0.1_ exposure leads to a characteristic asthma phenotype in offspring. Hamada et al. [78] initially demonstrated pregnant Balb/c mice exposed to aerosolized residual oil fly ash, a surrogate for ambient PM_2.5_, just prior to delivery significantly increased offspring OVA-induced airway hyperresponsiveness (AHR) and elevated levels of eosinophils in bronchoalveolar lavage fluid (BALF) driven by a skewed Th2 response. A primary feature of asthma involves a heightened inflammatory cell influx, largely composed of eosinophils or neutrophils driven via increased Th2 CD4+ T-cell signaling through interleukins (IL) 4 and 5 and/or Th17 signaling via IL-17. IL-4 also drives allergen-specific immunoglobin (Ig) production. Additionally, IL-13 elicits effects related to airway hyperresponsiveness (i.e., bronchoconstriction). Hamada and colleagues further highlight histopathology showing marked pulmonary inflammation and increased allergen-specific IgE and IgG levels. Fedulov et al. [83] subsequently showed pregnant Balb/c mice exhibit an acute inflammatory response to both diesel exhaust PM_0.1_ and more “inert” particles, titanium dioxide (TiO_2_), in contrast to non-pregnant females. Offspring born to mothers exposed to either type of particle went on to develop AHR and display airway inflammation following OVA challenge. These data underscore the state of pregnancy itself affects the host-response to particle exposure and confirm particles increase asthma susceptibly in offspring. In another model of prenatal exposure to diesel exhaust (DE), Corson et al. [82] co-exposed pregnant Balb/c mice to *Aspergillus fumigatus* allergen and DE. Offspring were challenged with this allergen at 9-10 weeks of age. Interestingly, offspring in the prenatal DE + allergen exposure group had decreased IgE production and dampened airway eosinophilia indicating potential protection against allergen-induced inflammation. These results emphasize the influence of co-exposures during gestation on asthma risk. Additionally, Sharkhuu et al. [102] demonstrated prenatal DE exposure altered some baseline inflammatory indices in the lung, which varied based on sex, but changes in response to OVA-induced inflammation were not significant in exposed mice. Manners et al. [84] combined several of the dosing strategies from previously described models to investigate the mechanisms underlying prenatal PM_2.5_ exposure and offspring asthma risk. Researchers showed repeated exposure to DEPs during gestation led to significant OVA-enhanced inflammation and AHR in offspring. Moreover, asthma susceptibility was associated with expression of genes regulated through oxidative stress and aryl hydrocarbon receptor (AhR) pathways. Diesel exhaust PM contains a mixture of polycyclic aromatic hydrocarbons (PAHs), known to upregulate AhR-related genes. The role of AhR in the immune response, including the production of Th2 and Th17, continues to evolve [109].

**Table 3:** Summary of respiratory and immune effects from in vivo models (17 studies)

Reference	Animal model	PM source	Dose	Route	Duration	Offspring effects
Hamada et al. [78]	Balb/c mice	Residual oily fish ash	50 mg/mL	Inhalation	30 min for 5, 3, or 1 days prior to delivery	Increased airway hyperresponsiveness and elevated levels of eosinophils in BALF; histopathology showed inflammation in lungs and increased IgE and IgG1 antigen-specific antibodies; Th2-skewed immune response
Fedulov et al. [78]	Balb/c mice	Diesel exhaust	50 μg/mouse (DEP, CB, or TiO_2_)	Intranasal	Single nasal insufflation on GD14	Airway hyperresponsiveness and airway inflammation, suggesting increased susceptibility to asthma
Mauad et al. [53]	Balb/c mice	PM_2.5_	16.8 +/− 8.3 μg/m^3^	Inhalation	4 months (24 h/day)	Decreased surface to volume ratio; reduced inspiratory and expiratory volumes
Corson et al. [82]	Balb/c mice	Diesel exhaust particles	Average particle concentration 1.09 mg/m^3^.	Inhalation	5 times 4 days apart (before mating), then 2 times after mating	Decrease in IgE production; decrease in pulmonary eosinophils
Sharkhuu et al. [102]	Balb/c mice	Diesel exhaust	0, 0.8, 3.1 mg/μL	Inhalation	10 consecutive days (4 h/day)	Number of neutrophils in BALF and splenic T cells expressing different surface markers were differentially affected by DE concentrations and sex; female offspring exposed to DE prenatally exhibited higher protein levels in the respiratory tract compared to males
Reiprich et al. [103]	Balb/c mice	Diesel exhaust particles	20 μg/mouse	Inhalation	GD7 to delivery (10 min 3 times/week)	Increased airway hyperresponsiveness, eosinophilic infiltration, and antigen-specific IgE production; pretreatment with N-acetylcysteine reversed asthmatic phenotype in pollutant-exposed offspring
Thevenot et al. [72]	C57BL/6J mice or Brown-Norway rats	EPFR (DCB-230) in combustion-generated PM (0.2 μm)	200 μg/m^3^	Inhalation	7 days (30 min/day) neonatal exposure only	Increased pulmonary pathologies and development of asthma; increased airway hyperresponsiveness
Wang et al. [104]	C57BL/6J mice	CB-NP (unknown diameter) + MCP-230	50 μg/kg BW	Oropharyngeal	GD10 and GD17 (once/day)	MCP-230 exposed dams, offspring have decreased production of T helper and Tregs; Th1 and Th17 cells remained
Manners et al. [84]	C57BL/6J mice	Diesel exhaust particles	50 μg/mouse	Intranasal	GD3, 6, 9, 12, 15, 18 (once/day)	Increase airway inflammation and hyperresponsiveness; increase OVA-specific IgE
Saravia et al. [73]	C57BL/6J mice	CDPM	200 μg/m^3^	Inhalation	5 days (30 min/day) neonatal exposure only	CDPM+HDM challenged mice exhibited no noticeable asthma phenotype, AHR, Th2 inflammation, eosinophilia, HDM Ig-specific; CDPM induce immunosuppressive environment; CDPM suppression of Th2 response
Lee et al. [74]	C57BL/6J mice (neonates < 7-day age)	CDPM (0.2 μm); EPFR (DCB-50 and DCB-230)	200 μg/m^3^	Inhalation	5 days (30 min/day)	Enhanced morbidity and decreased survival; increased oxidative stress; increased pulmonary Tregs during influenza
El Sayed et al. [105]	ICR mice	CB-NP (14 nm diameter)	95 μg/kg BW	Intranasal	GD9 and GD15 (once/day)	Increased total thymocytes and immunophenotypes; increase total lymphocytes in male offspring at PND5 exposed to CBNP; upregulation of mRNA expression of genes involved with induction of peripheral tolerance
Paul et al. [106]	C57BL/6J mice	TiO_2_, CeO_2_, Ag nanoparticles (10 nm diameter)	10 μL of NPs at 10 mg/mL	Intratracheal	GD2.5, GD9.5, and GD16.5	Offspring lung development was stunted regardless of nanoparticle exposure
Jaligama et al. [76]	C57BL/6J mice	CDPM (0.2 μm); EPFR (DCB-230)	200 μg/m^3^	Inhalation	7 days (30 min/day) neonatal exposure only	Increase in Tregs and IL-10; EPFR+ IL-10−/− neonates exhibited reduced morbidity, viral load, and adaptive T cell response after influenza infection
de Barros et al. [107]	Balb/c mice	PM_2.5_	600 μg/m^3^	Inhalation	GD5.5-GD18.5 (1 h/day)	Increased lung elastance and decreased alveolar number in PND40 offspring; lung volume and BALF were not affected; transcriptomic analysis indicated DNA damage, inflammation, and cell proliferation regulation in PND40 exposed offspring
Tang et al. [94]	Sprague Dawley rats	PM_2.5_	0.1, 0.5, 2.5, 7.5 mg/kg	Intraperitoneal	GD0-GD18 (once every 3 days)	Ground glass opacity and high-density volumes in lungs of exposed neonates; increased pulmonary inflammation in lungs
Rychlik et al. [68]	C57BL/6J and Balb/c mice	Ultrafine PM	100 μg/m^3^	Inhalation	GD0-GD18 (6 h/day)	Reduced inflammatory response to HDM; lower WBC counts; less peribronchiolar inflammation; C57Bl/6J offspring exposed to UFPs had increased Treg response and decreased Th2/Th17 response

*BALF* bronchoalveolar lavage fluid, *DE* diesel exhaust, *HDM* house dust mite, *UFPs* ultrafine particles, *GD* gestational day, *CDPM* combustion-derived PM, *EPFR* environmentally persistent free radicals

Asthma is a complex chronic disease with multiple elements involved in its etiology, including genetic predisposition and a variety of environmental factors. Maternal exposure to microbial-rich environments is suggested to play a protective role against childhood asthma and allergy development [110]. Using an innovative approach, Reiprich et al. [103] tested the ability of endotoxin (lipopolysaccharide, LPS), representative of microbial exposure, to protect against offspring asthma development. LPS protected against OVA-induced pulmonary inflammation and AHR; however, in offspring prenatally exposed to DEPs, LPS failed to confer protection. Investigators showed the protection was dependent on the epigenetic regulation of IFNγ expression. Maternal supplementation with the antioxidant N-acetylcysteine reversed these effects suggesting that maternal dietary supplementation may serve as a preventive intervention to combat DEP-induced oxidative stress and downstream consequences in offspring.

Structural alterations in lung development and related functional changes have also been intensely investigated in rodent models of prenatal fine and ultrafine PM exposure. Mauad et al. [53] revealed offspring exposed pre- and postnatally to heavy traffic in São Paulo presented smaller surface to volume ratios and decreased inspiratory and expiratory volumes. Subsequent research by this group substantiates these structural and functional defects [107]. While glandular and saccular structures of fetal lungs were not substantially altered following gestational exposure to concentrated urban PM_2.5_ and PM_0.1_ from São Paulo, offspring showed significantly lower alveolar number and higher lung elastance at postnatal day 40. Genes related to DNA damage, cell proliferation, and inflammation were differentially expressed in the fetal lung suggesting a complex interplay of pathways influencing long-term lung alterations. Research on prenatal PM_0.1_ exposure mirrors work related to manufactured nanoparticles. Paul et al. [106] showed gestational exposure to titanium dioxide (TiO_2_), cerium oxide (CeO_2_), and silver nanoparticles-induced stereotyped impairment of lung development (decreased radial alveolar count/alveolar surface) with lasting effects in adult mice. These effects were independent of the chemical nature of the nanoparticles indicating particle size played a primary role.

In a rat model, repeated gestational PM_2.5_ exposure resulted in significant changes in offspring lung structure and function, including increased lung consolidation, airway inflammation, and decreased lung volume and compliance [94]. Additionally, in PM_2.5_-exposed offspring, investigators observed interstitial proliferation, significant oxidative stress in lungs, and upregulation of epithelial-mesenchymal transition (EMT). EMT is a process where fully differentiated epithelial cells undergo transition to a mesenchymal phenotype, thus losing typical epithelial markers like E-cadherin. Transforming growth factor-beta (TGF-β), a key mediator of EMT, can be influenced by oxidative stress/reactive oxygen species (ROS), suggesting the link between PM_2.5_ and aberrant ROS signaling underlying the process of EMT [111]. This phenotype was also characterized in a mouse model of neonatal exposure to radical-containing ultrafine particles [72]. Investigators proposed EMT in neonatal mouse lungs following acute exposure to ultrafine particles may underlie epidemiological evidence supporting PM exposure and increased risk of asthma. Successive research from this group supports the role of early life PM_0.1_ exposure and increased risk of both asthma and respiratory infection risk. Saravia et al. [73] demonstrated an early immunosuppressive phenotype in mice exposed to PM_0.1_ and challenged with house dust mite (HDM). Herein, the PM-HDM group failed to develop the typical asthma-like phenotype; however, offspring developed an allergic phenotype upon re-challenge later in life. The “switching” observed in this study indicates the crucial importance of the timing of exposure, as well as when pulmonary assessment is conducted. Moreover, the initial immune suppression is relevant to respiratory infection risk, which investigators went on to show neonates exposed to PM_0.1_ early in life were more susceptible to severe influenza infection [74]. Likewise, in a mouse model of prenatal exposure to PM_0.1_, Rychlik et al. [68] demonstrated a dampened response to HDM challenge in offspring from the exposed group. The role of altered adaptive immune response appears to underlie the muted response. Circulating IL-10 was significantly upregulated in offspring exposed to PM_0.1_, suggesting increased regulatory T cell (Tregs) expression and suppressed Th2/Th17 response. Jaligama et al. confirmed the role of IL-10 and Tregs in suppressing the adaptive response following early life PM_0.1_ exposure [76]. Depletion of Tregs reduced morbidity and conferred enhanced protection against influenza virus.

El-Sayed et al. [105] also demonstrated gestational exposure to carbon black nanoparticles induced dysregulation of lymphocyte populations in offspring, indicating neonatal peripheral tolerance. These effects could be predictive of allergic or inflammatory responses in childhood. Importantly, the magnitude of alteration depended on the stage of gestation fetuses were exposed, highlighting the importance in the timing and duration of exposure. In support of the hypothesis that prenatal PM alters immune development and predisposes offspring toward asthma, Wang et al. [104] showed gestational exposure to combustion-derived PM_0.1_ inhibited offspring pulmonary T cell development, with suppression of Th1, Th2, Th17, and Tregs at 6 days of age. Pulmonary Th1 cells remained suppressed up to 6 weeks, leading to enhancement of postnatal allergic responses to OVA evidenced by increased AHR, eosinophilia, and pulmonary Th2 responses. Overall, the bulk of the non-human evidence validates prenatal PM_2.5_ and PM_0.1_ exposure alter offspring lung and immune system development, signifying risk for acute (respiratory infection) and chronic (asthma) pulmonary health outcomes. Since variations in fetal development of lung and immune system vary between humans and rodents, it is sometimes difficult to interpret the translational relevance in regards to trimester-specific effects. Nonetheless, combined evidence from human and non-human studies support reducing exposure may prevent the tremendous burden of respiratory morbidity in infants and children.

### Cognitive effects

The neurodevelopmental effects of prenatal PM exposure are well-documented in numerous epidemiological studies. Likewise, in the past 10 years, the number of animal studies has expanded considerably verifying the adverse cognitive effects and underlying neurotoxic mechanisms (Table 4). In numerous models, prenatal exposure to fine and ultrafine PM leads to offspring cognitive and behavioral impairment, often in a sex-specific manner bias toward effects on male offspring. Neurodevelopmental disorders, including autism spectrum disorder (ASD), have an increased prevalence in males. Boys are approximately three times more likely to be diagnosed than girls [119]. Findings from laboratory studies highlight how PM-induced neurological damage, including structural and functional changes, are region, sex, and timing-dependent.

**Table 4:** Summary of cognitive effects from in vivo models (19 studies)

Reference	Animal Model	PM Source	Dose	Route	Duration	Offspring Effects
Suzuki et al. [112]	ICR mice	Diesel exhaust	171 μg/m^3^	Inhalation	GD2-GD16 (8 h/day)	Decreased spontaneous locomotor activity; neurotransmitters (dopamine and noradrenaline) and metabolites were increased in the prefrontal cortex; decreased SLA due to facilitated release of dopamine in the PFC
Allen et al. [55]	C57BL/6J mice	Ultrafine PM, concentrated ambient particles	∼40,000-496,000 particles/cm^3^	Inhalation	PND4–7 and PND10–13 (with and without adult exposure over PND56–60)	Increased FR response rates and FR resets, as well as enhanced bias for immediate reward.
Davis et al. [79]	C57BL/6J mice	nPM; < 200 nm	350 μg/m^3^	Inhalation	5 h/day 3 days/week for 10 weeks (females exposed 7 weeks before conception to 2 days before birth)	Impaired cerebral cortex neuron development in vitro; increased depression-like phenotype in tail-suspension test (male); additional nPM exposure promoted pyramidal neuron development
Allen et al. [56]	C57BL/6J mice	Ultrafine PM, concentrated ambient particles	∼40,000 to 496,000 particles/cm^3^	Inhalation	PND4–7 and PND10–13 (with and without adult exposure over PND57–59)	Impaired short-term memory on the NOR; mechanistically, cortical and hippocampal changes in amino acids raised the potential for excitotoxicity, and persistent glial activation in frontal cortex and corpus callosum of both sexes
Allen et al. [57]	C57BL/6J mice	Ultrafine PM, concentrated ambient particles	96 μg/m^3^	Inhalation	PND4–7 and PND10–13 (with a group of males getting additional exposure on PND270)	Ventriculomegaly in males that persisted through young adulthood; males also showed a decrease in CNS cytokines (whereas females showed an increase); males exposed on PND270, showed changes in CNS neurotransmitters and glial activation across multiple brain regions and increased hippocampal glutamate
Yokota et al. [97]	ICR mice	Diesel exhaust	90 μg/m^3^	Inhalation	GD2-17 (8 h/day)	Increased social isolation-induced territorial aggressive behavior; increased serum testosterone levels; increased dopamine levels in prefrontal cortex and nucleus accumbens; lower serotonin in nucleus accumbens, amygdala, and hypothalamus
Onoda et al. [113]	ICR mice	CBNP	2.9, 15, 73 μg/mL	Intranasal	GD5 and GD9 (once/day)	Increase in GFAP in cerebral cortex; increase aquaporin-4 in brain parenchyma; increased expression of Flt1, Sox17, Tgfa, Cyr61
Cory-Slechta et al. [59]	C57BL/6J mice	Ultrafine PM, concentrated ambient particles	45 μg/m^3^	Inhalation	PND4–7 and PND10–13 (4 h/day)	Male-specific alterations in learning and memory functions, while females showed alterations in motivational behaviors but not final performance
Klocke et al. [61]	B6C3F1 mice	Ultrafine PM, concentrated ambient particles	92.69 μg/m^3^	Inhalation	GD0.5-16.5 (6 h/day)	Ventriculomegaly; increased corpus callosum area and reduced hippocampal area in both sexes; CC hypermyleination, microglial activation, and reduced total CC microglia in both sexes; increased iron deposition in CC of female offspring
Allen et al. [58]	C57BL/6J mice	Ultrafine PM, concentrated ambient particles	96 μg/m^3^	Inhalation	PND 4-7 and PND 10-13 (4 h/day for 4 days/week)	Induced inflammation/microglial activation; reduced size of corpus callosum; hypomyelination; aberrant white matter development; increased glutamate; increased repetitive and impulsive behavior
Kulas et al. [114]	FVB mice	PM_2.5_	46.7 μg/m^3^	Inhalation	Gestation (6 h/day 5 days/week)	Deficits in spatial memory; long-term inflammation and neurodegeneration through increased levels of COX2; changes in levels of synaptophysin and Arg1 proteins; changes in the concentration of cytokines in the brain and spleen
Klocke et al. [62]	B6C3F1 mice	Ultrafine PM, concentrated ambient particles	92.69 μg/m^3^	Inhalation	GD0.5-16.5 (6 h/day)	Elevated iron levels in the cerebellum of females; altered cerebellar gene expression; significant enrichment in inflammation and transmembrane transport processes
Kloche et al. [115]	B6C3F1 mice	Ultrafine PM, concentrated ambient particles	92.69 μg/m^3^	Inhalation	GD0.5-16.5 (6 h/day)	Premature maturational shift in number and proportion of total cells and hypermethylation in both sexes; ventriculomegaly in females (possible amelioration of ventriculomegaly in males); alterations in cycling Ki67+/Olig2+ cell number and proportion of total cells in the female corpus collosum; total CC cellularity was slightly elevated in males
Woodward et al. [116]	Sprague Dawley rats	TRAP	240 μg/m^3^	Inhalation	GD2, through gestation, until 25 weeks of age (5 h/day, 3 days/week)	At 5 month males had 70% fewer newly generated neurons in the dentate gyrus (DG) of the hippocampus; microglia were activated in DG/CA1 subfields (35% more Iba1); altered blood-brain-barrier, with a 75% decrease of the tight junction protein ZO-1 in the CA1 layer, and twofold more iron deposits, a marker of microhemorrhages; impaired contextual memory, reduced food- seeking behavior, and increased depressive behaviors
Sobolewski et al. [60]	C57BL/6J mice	Ultrafine PM, concentrated ambient particles	45 μg/m^3^	Inhalation	PND4–7 and PND10–13 (4 h/day)	Decreased serum T concentrations; social nose-to-nose sniff rates with novel males in adulthood; adult T serum concentrations were positively correlated with nose to nose sniff rates
Chang et al. [98]	C57BL/6J mice	Diesel exhaust	250-500 μg/m^3^	Inhalation	GD0 to PND21	Deficits in all three of the hallmark categories of ASD behavior: reduced social interaction, increased repetitive behavior; reduced or altered communication
Church et al. [63]	B6C3 mice	Ultrafine PM, concentrated ambient particles	135.8 μg/m^3^	Inhalation	Gestation followed by additional exposures to both dams and their litters from PND2-10	Significantly decreased sociability in both sexes; however, reductions in reciprocal social interaction/increased grooming behavior were only present in males
Cui et al. [117]	ICR mice	Diesel exhaust particles	0.4 mg/m^3^	Inhalation	GD1.5-GD15.5 (6 h/day)	Longer total distance and time in open field test; increased expression of dopamine receptors (Drd1a, Drd2, Drd3, Drd4, Drd5)
Morris-Schaffer et al. [118]	C57BL/6J mice	Ultrafine elemental carbon	50 μg/m^3^	Inhalation	PND4–7 and 10–13 (4 h/day)	No significant difference in anogenital distance, BW, or CNS pathological; no changes in novel object recognition; elevated plus maze performance, FI, or DRL schedule-controlled behavior

*GD* gestational day, *PND* postnatal day, *FR* fixed ratio, *NOR* novel object recognition, *CC* corpus callosum

Findings from Cory-Slechta and colleagues demonstrate early postnatal exposure to concentrated ambient ultrafine PM altered behavioral responses related to impulsivity, with exposed mice showing a preference for immediate reward [55]. This window of exposure is critical to central nervous system development and equivalent to human 3rd trimester brain development [51]. Following exposure, both sexes showed impaired learning and short-term memory outcomes and persistent glial activation in the frontal cortex and corpus callosum [56]. Exaggerated microglial activation and accompanying inflammation can have detrimental effects on neurodevelopment [120]. Increased expression of the glial fibrillary acidic protein (GFAP), a marker of activated astrocytes, has consistently been correlated with developmental PM_2.5_ and PM_0.1_ exposure [113, 114]. Additional phenotypes related to impulsivity measured using fixed interval (FI) schedule-controlled performance tests reveal varied results based on sex and timing of exposure [56]. Changes in dopamine and glutamate systems known to mediate FI performance coincided with phenotypic changes. Findings related to altered locomotor effects also signify the role of dopamine pathway activation, as well as glycine signaling inhibition [112, 117]. Supplementation with a dopamine receptor antagonist and glycine receptor agonist attenuated adverse effects [117].

Overall, sex-specific behavioral changes are consistently observed. Male-specific learning/memory-related deficits have occurred even at low level neonatal ultrafine PM exposure, whereas female neonates have displayed altered motivational behaviors but not changes in overall performance [59]. Allen et al. [57] verified early postnatal PM_0.1_ exposure led to ventriculomegaly, i.e., lateral ventricle dilation, preferentially in male mice that persisted through young adulthood. In complementary work, Klocke et al. [61] investigated the impact of in utero ultrafine PM exposure on offspring neurodevelopment. Exposure to concentrated ambient ultrafine particles from gestation days 0.5-16.5 also resulted in ventriculomegaly, increased corpus callosum (CC) area and reduced hippocampal area in male and female offspring. Both sexes demonstrated increased microglial activation and reduced total CC microglia number and CC hypermyelination. CC iron (Fe) deposition was increased in female offspring. Altered Fe deposition can underlie oxidative stress and indicate pathologic conditions [121]. Subsequently, Klocke et al. [62] observed a similar increase in cerebellum Fe concentrations in female offspring exposed prenatally. Iron, as well as aluminum and silicon inclusions, also presented in the CC with ultrastructural myelin sheath damage. Long-term myelin status assessed at a stage of early brain maturity revealed persistent hypermyelination in PM-exposed offspring of both sexes [115]. Findings from Woodward et al. [116] confirm glial activation and accumulation of Fe deposits. In their model, rats were exposed ultrafine PM < 200 nm diameter collected near a Los Angeles freeway throughout gestation into adulthood. Investigators only followed male offspring, and at 5 months of age observed microglia activation and increased Fe deposition. Exposed animals showed behavioral deficits, including increased depressive behavior.

Depressive behavior, aggression, and behavioral features of autism spectrum disorder (ASD) are documented across several rodent models. Davis et al. [79] exposed mice to PM_0.1_ collected from an urban freeway (as detailed above) prior to mating and throughout gestation. Male offspring displayed increased depression-like responses. Additionally, Yokota et al. [97] observed male offspring exposed to low level DE during the prenatal period had significantly greater social isolation-induced territorial aggressive behavior in comparison to control mice. This behavior correlated with higher serum testosterone levels in exposed males. Likewise, dopamine levels were higher in the prefrontal cortex and nucleus accumbens, a functional part of the reward system, whereas serotonin levels were lower in the nucleus accumbens, amygdala, and hypothalamus in socially isolated DE-exposed mice. Sobolewski et al. [60] corroborated some of these male-biased hormonal and neurochemical changes in a model of early postnatal ultrafine PM exposure. Male offspring had lower serum testosterone levels following exposure and showed male social novelty preference, suggesting social communication deficits. Chang et al. [98] demonstrated mice exposed to diesel exhaust throughout gestation exhibited deficits in three of categories characteristic of ASD-related behavior, including reduced social interaction, increased repetitive behavior, and reduced/altered communication. Church et al. [63] corroborated these effects linking early life PM_2.5_ exposure with an ASD phenotype. Mice exposed to fine PM during gestation and the early neonatal period displayed reduced sociability in both sexes and increased repetitive deficits in male offspring. In a model of early postnatal ultrafine PM exposure, Allen et al. [58] observed a pattern of developmental neurotoxicity aligned with the mechanistic underpinnings of ASD. PM-exposed offspring displayed neuroinflammation, microglial activation, reduced CC area and associated hypomyelination, aberrant white matter development, ventriculomegaly, elevated glutamate and excitatory/inhibitory imbalance, increased amygdala astrocytic activation, and repetitive and impulsive behaviors, many endpoints male-biased. Collectively, these findings along with structural and functional changes highlighted above emphasize the vulnerability of the developing brain to early life fine and ultrafine PM exposure. Studies teasing apart the critical components of PM responsible for adverse cognitive effects highlight the role of reactive metals, as well as PAHs [122], in eliciting pathology and behavioral dysfunction, versus the elemental carbon fraction alone [118].

### Cardiometabolic effects

Accumulating evidence supports an impact of prenatal fine and ultrafine PM exposure on diverse metabolic diseases in offspring, such as diabetes and obesity, as well as adverse effects on the cardiovascular system. Cardiac dysfunction precedes overt heart failure, and classic risk factors include high blood pressure (hypertension), heart attack (myocardial infarction), enlargement of the heart (cardiomyopathy), and diabetes. While well-established in epidemiologic studies of adults and elderly exposed to fine PM [123], evidence of cardiac dysfunction following developmental exposure to PM_2.5_ and PM_0.1_ is only now emerging [42] (Table 5). The fetal origins of coronary disease are well-recognized by the “Barker hypothesis,” wherein fetal undernutrition manifesting initially as intrauterine growth retardation/low birth weight precedes hypertension, coronary heart disease, and non-insulin-dependent diabetes in adulthood [126]. Since 2013, several rodent models published support the findings that prenatal PM_2.5_ and PM_0.1_ exposures cause offspring cardiac dysfunction and heart disease risk later in life.

**Table 5:** Summary of cardiometabolic effects from in vivo models (13 studies)

Reference	Animal model	PM source	Dose	Route	Duration	Offspring effects
Weldy et al. [99]	C57BL/6J mice	Diesel exhaust PM_2.5_	300 μg/m^3^	Inhalation	3 weeks prior to mating; duration same through gestation (6 h/day, 5 days/week)	Increased susceptibility to cardiac hypertrophy, systolic failure, myocardial fibrosis, pulmonary congestion
Gorr et al. [64]	FVB mice	PM_2.5_	51.69 μg/m^3^	Inhalation	GD0-PND21 (6 h/day, 7 days/week)	Reduced birth weight; reduced left ventricular fractional shortening; reduced ejection fraction; increased end-systolic volume; reduced dP/dt maximum; reduced peak shortening; slower calcium reuptake; reduced response to B-adrenergic stimulation; increased collagen deposition
Wei et al. [54]	Sprague Dawley rats	Ambient air	73.5 +/− 61.3 μg/m^3^	Inhalation	19 days (24 h/day)	Increased body weight; peribronchiolar and perivascular inflammation; increased systemic and oxidative stress; dyslipidemia; increased inflammatory status of epididymal fat
Stephenson et al. [75]	C57BL/6J mice	Ultrafine PM MCP-230	50 μL	Oropharyngeal	GD10 and GD 17 (once/day)	Reduced skeletal muscle mtDNA copy number; lower mRNA levels of electron transport genes; reduced citrate synthase activity; upregulation of genes in reducing oxidative stress in muscle
Tanwar et al. [65]	FVB mice	PM_2.5_	73.61 μg/m^3^	Inhalation	Gestation (6 h/day, 7 days/week)	Reduced fractional shortening; increased left ventricular end-systolic and end-diastolic diameters; reduced ventricular posterior wall thickness; end-systolic elastance; contractile reverse; increased collagen deposition; increases in cardiac IL-6, IL-1B, collagen-1, MMP9, and MMP13 gene expression
Goodson et al. [124]	C57BL/6J mice	Diesel exhaust	300 μg/m^3^	Inhalation	Gestation (6 h/day, 5 days/week)	Dysregulated gene expression of Mir133a-2, Ptprf, Pamr1; promoter of Mir133a-2 differentially methylated
Harrigan et al. [100]	C57Bl/6 (ApoE−/−) mice	Diesel exhaust particles PM_2.5_	250-300 μg/m^3^	Inhalation	Gestation (6 h/day, 5 days/week)	Smaller litter and increased postnatal mortality; no significant differences in plasma lipids or lipoprotein profiles, expression of antioxidant genes or markers of oxidative stress between groups; no sig. differences in atherosclerotic lesion area
Chen et al. [86]	C57BL/6J mice	Diesel exhaust particles PM_2.5_	160 μg/m^3^	Intratracheal	7-week preconception period, whole gestation, and lactational periods (3 times/week)	Impaired adult male offspring glucose tolerance; no influence in insulin sensitivity for adult male offspring; decreased insulin secretion; decrease in pancreatic islet and Beta cell sizes
Ye et al. [125]	Sprague Dawley rats	PM_2.5_	1 mg/kg BW	Oropharyngeal	GD8, 10, 12 (once/day)	Increased blood pressure; impaired sodium excretion; reduced D1R-mediated natriuresis and diuresis; increased D1R and GRK4 expression
Wu et al. [69]	Sprague Dawley rats	Ultrafine PM (10-20 nm diameter; ammonium sulfate particles)	150 μg/m^3^	Inhalation	GD0-GD18	Increased stillbirths; reduced gestation length and birth weight; increased concentrations of glucose and free fatty acids in plasma; increased lipid accumulation in liver; decreased endothelium-dependent relaxation of aorta
Morales-Rubio et al. [92]	C57BL/6J mice	Ultrafine PM (< 60 nm)	50 μL UFP followed by 200 μL air (400 μg/kg BW)	Intratracheal	GD6.5, 8.5, 10.5, 12.5, 14.5, 16.5 (once/day)	Increased embryo reabsorption; decreases in uterus, placental, and fetal weights; HSD11B2 hypermethylation and protein downregulation; increases in PAH enzymes; activation of AT1R and ACE in PND50 resulting in increased blood pressure
Woodward et al. [80]	C57BL/6J mice	Ultrafine PM (< 100 nm)	343 μg/m^3^	Inhalation	Gestation (5 h/day, 3 days/week)	Increased food intake, body weight, fat mass, adiposity, glucose intolerance; dysregulation of neuropeptides in hypothalamus; decreased expression of insulin receptor signaling genes in adipose
Xie et al. [90]	C57BL/6J mice	PM_2.5_	0.3 mg/kg/day	Oral	GD0-GD18 (once/2 days)	Reduced BW in adult males after 6 weeks; reduced adipocytesize in eWAT compared to BAT; decreased expression of ACC1, ACSL1, PPAR-a in eWAT; decreased expression of TNF-a, IL-1B, IL-6; reduction of LPC, PC, PE, SM, Cer.

*GD* gestational day, *BW* body weight

Weldy et al. [99] exposed C57Bl/6 mice to filtered air or diesel exhaust (DE) prior to mating for 3 weeks, during gestation, and until offspring were 8 weeks of age. At 12 weeks of age, male offspring underwent a transverse aortic constriction (TAC) surgery to induce pressure overload. Exposed mice showed increased risk of cardiac hypertrophy, systolic failure, myocardial fibrosis, and pulmonary congestion following TAC surgery, indicative of risk for heart failure. A study by Gorr et al. [64], in which investigators exposed FVB mice to PM_2.5_ during pregnancy and continuing until time of weaning concluded that early life exposure to fine PM leads to cardiac dysfunction. At adulthood, exposed mice showed reduced left ventricular fractional shortening, with greater left ventricular end-systolic diameter. Pressure-volume loops revealed alterations in several key parameters associated with dysfunction. Histological analyses highlighted increased cardiac collagen deposition, a precursor to fibrosis. Tanwar et al. [65] further clarified the mechanisms of heart failure susceptibility following in utero exposure to PM_2.5_. Investigators demonstrated several adverse phenotypes in exposed offspring evaluated at 12 weeks, including reduced fractional shortening, increased left ventricular end-systolic and end-diastolic diameters, reduced left ventricular posterior wall thickness, end-systolic elastance, contractile reserve, frequency-dependent acceleration of relaxation, and blunted contractile response to β-adrenergic stimulation. Moreover, histological assessment showed increase collagen deposition in exposed offspring, confirming findings from Gorr et al. [64, 65]. Acute inflammatory markers, alterations in Ca^2+^ handling proteins, and changes in protein expression of DNA methyltransferases were also marked in the exposed group, suggesting the role of epigenetic changes in priming heart disease risk. Goodson et al. [124] subsequently demonstrated in utero exposure to DE altered DNA methylation in cultured neonate cardiomyocytes.

Additional studies have investigated the impact of developmental fine and ultrafine PM exposure on blood pressure and vessel vasodilatory response. Wu et al. [69] showed adult Sprague-Dawley rats exposed to ultrafine PM throughout gestation had impaired relaxation of the nitric oxide (NO)-mediated vasodilatory response of their aortas. A hallmark of cardiovascular dysfunction is a reduced bioavailability of NO, a major vasodilator, from endothelial cells [127]. PM-driven reactive oxygen species (ROS) can react with NO to yield reactive peroxy species (e.g., peroxynitrite), and this reaction leads to both a NO deficiency and a depletion of reduced glutathione (the major antioxidant in cells), constituting a vicious cycle [128]. Morales-Rubio et al. [92] further showed in utero exposure to ultrafine PM induced placental stress in mice, via intrauterine oxidative damage and inflammation, stimulating programming and activation of the angiotensin II receptor type 1 (AT_1_R) and angiotensin I-converting enzyme (ACE) in offspring lung. These changes correlated with increased blood pressure in male offspring around 7 weeks of age. Ye et al. [125] investigated the role of the regulation of renal sodium transport in increased blood pressure following in utero exposure to fine PM. In this study, Sprague-Dawley rats were repeatedly exposed to PM_2.5_ during pregnancy; exposed offspring increased offspring blood pressure and decreased renal G protein-coupled receptor kinase type 4 (GRK4) expression, a receptor that plays an important role in sodium transport. Furthermore, exposed offspring had impaired renal dopamine D1 receptor-mediated sodium excretion. Strikingly, these abnormalities were normalized by administration of the antioxidant tempol, suggesting antioxidants may serve as potential therapeutics for PM-mediated hypertension.

While these studies signify the potential for developmental PM_2.5_ and PM_0.1_ exposure to influence hypertension, a major risk factor for heart disease, another risk factor, atherosclerosis, was not shown to be a consequence of prenatal exposure in a recent model. Harrigan et al. [100] exposed pregnant apolipoprotein E-deficient mice (Apoe^−/−^) to DE throughout gestation. The Apoe^−/−^ mouse model is useful for studying cardiovascular disease since mice have poor lipoprotein clearance, which supports cholesterol accumulation in blood, thereby promoting the development of atherosclerotic plaques [129]. While there was higher postnatal mortality in the DE-exposed mice, there were no significant differences in plasma lipids or lipoprotein profiles, expression of antioxidant genes or markers of oxidative stress between treatment groups at 16 weeks of age. DE-exposed offspring did present a higher frequency of atherosclerotic lesions, but overall, there were no significant difference in average atherosclerotic lesion area in the aortic sinus or innominate arteries. Likewise, Wu et al. [69] did not observe differences in concentrations of non-esterified fatty acids in plasma of male and female rats at 3 weeks of age following prenatal exposure to ultrafine PM or filtered air. Paradoxically, investigators measured significant decreases in plasma triacylglycerol concentrations in PM_0.1_-exposed offspring (both male and female). Wei et al. [54] chronically exposed rats to either filtered or unfiltered polluted Beijing air throughout gestation to 8 weeks of age. Blood markers showed exposure to unfiltered air resulted in a worsened lipid profile, reduced GLP-1 levels, an incretin hormone that reinforces glucose-dependent secretion of insulin, and reduced antioxidant capacity parallel with measures of increased oxidative stress. Additionally, epididymal fat mass was greater in the male offspring exposed to polluted air. Overall, these findings based on three studies (in different models with distinct exposure durations and varying pollutants) necessitate additional mechanistic studies to determine the role of developmental fine and ultrafine PM exposures on plasma lipid profiles. However, findings from Wei et al. [54] related to effects on glucose homeostasis and fat accumulation are substantiated by additional studies detailed below.

Several metabolic disease states are characterized, including diabetes phenotypes (i.e., disruption of glucose metabolism) and obesity risk. Largely, studies show prenatal PM_2.5_ and PM_0.1_ exposure can alter glucose homeostasis in offspring, in particular male offspring. Chen et al. [86] demonstrated prenatal exposure to DE PM_2.5_ in C57Bl/6 mice altered the morphology and function of male offspring pancreatic β cells, which are responsible for producing insulin, thus altering glucose metabolism. This deficiency correlated with decreased pancreatic islet cell and β cell sizes. Woodward et al. [80] observed a sex-specific impairment of glucose tolerance in C57Bl/6 male offspring exposed to PM_0.1_ during gestation through 17 weeks of age. These changes correlated with increased body weight, fat mass, adiposity, and increased food intake, representing an altered feeding behavior. Underlying these effects were significant changes in expression of metabolically relevant neuropeptides in the hypothalamus and decreased expression of insulin receptor signaling genes in adipose tissue. Male susceptibility was further confirmed in a study by Xie et al. [90], although in this model offspring exhibited sustained growth inhibitory effects, and not obesity. Following prenatal exposure to PM_2.5_, male mice showed reduced body weight beginning at 6 weeks of age, along with decreased epididymal adipose tissue, where there was a concurrent downregulation of genes related to fatty acid synthesis and oxidation. Lipidomic analysis revealed disruptions in sphingomyelin-ceramide signaling and glycerophospholipids remodeling. Notably, investigators did not carry out this analysis in female offspring since no changes in body weight were observed over the 5- to 15-week postnatal growth period.

These data mirror the varying growth effects observed in epidemiologic and animal studies, where different models report differing impacts on longitudinal growth in offspring. Indeed, Chen et al. [66] reported obesity in male offspring following prenatal exposure to PM_2.5_ in their mouse model employing concentrated ambient particle (CAP) exposure. Here, male offspring showed increased food intake, but were sensitive to exogenous leptin, a hormone involved in the regulation of energy intake and expenditure [130]. Leptin promoter methylation in adipocytes was significantly increased in PM_2.5_-exposed males but not females, signifying the role of altered leptin in programing male obesity. Stephenson et al. [75] reported that male mice exposed in utero to combustion-derived PM_0.1_ had increased weight gain in comparison to controls after placement on a high-fat diet for 12 weeks (from 10-22 weeks of age). Increased body weight was not associated with increased food intake, but correlated with lessened physical activity and energy expenditure. This corresponded with reductions in skeletal muscle mitochondrial DNA copy number, lower expression of electron transport genes, and reduced citrate synthase activity. Although the mechanisms behind these changes remain to be determined, accumulating findings that prenatal fine and ultrafine PM exposure can alter glucose metabolism later in life highlights a need for further research in this area, especially based on the current childhood obesity epidemic [30].

## Biological mechanisms underlying adverse effects

The range of adverse health outcomes in children following early life fine and ultrafine PM exposure are understood to be caused through two broad mechanisms, (1) directly crossing the placenta, hence, reaching fetal circulation and/or (2) indirectly through an interplay of PM-driven maternal/placental oxidative stress, inflammation, epigenetic modifications in placental and offspring tissues, and potential endocrine disruption [131, 132].

### Placental translocation of ultrafine particles

The placenta is a multifunctional organ that connects the developing fetus to the uterus through the umbilical cord. The primary function to provide oxygen and nutrients, remove waste products, and importantly, serve as a barrier between maternal and fetal tissues. Evidence from in vivo animal exposure models, ex vivo human placental models, and recent direct evidence from human placentae demonstrate transport of ultrafine particles (UFPs) across the placental barrier indicating possible direct effects on placental function and offspring health [133, 134].

In a rabbit model of diesel exhaust exposure, filtered to generate ultrafine PM (~69 nm diameter particles), Valentino et al. [135] observed transplacental transfer of particles using transmission electron microscopy (TEM) analysis. Nanoparticles were visualized in maternal blood spaces of exposed placentae, in trophoblastic cells, and within fetal vessels. Investigators also demonstrated disruption of placental function in exposed animals, which was largely due to reduced placental vascularization. Veras et al. [136] also reported functional morphologic changes in placentae, primarily on the maternal side, from mice exposed to ambient levels of air pollution in São Paulo. Exposure during gestation resulted in smaller fetal weights, reduced volumes, calibers, and surface areas of maternal blood spaces, and greater fetal capillary surfaces and diffusive conductance, which authors discussed as a fetoplacental adaption to maintain and expand oxygen and nutrient delivery. In another mouse model, Paul et al. [106] confirmed decreased placental efficiency with the presence of metallic nanoparticles in the placenta. This correlated with impaired lung development in offspring that persisted into adulthood.

Studies in human placentae have also verified placental translocation of ultrafine particles. Wick et al. [137] demonstrated the size-dependent transport of fluorescent polystyrene particles with diameters of 50, 80, and 240 nm (but not 500 nm) across human placental explants into the fetal circuit. Bové et al. [134] was the first research group to visualize particles on the fetal side of human placentae in a maternal cohort study. Black carbon (BC) particle aggregates ranged between 1.00 and 9.78 μm and consisted of various smaller particles that translocated from maternal circulation to distinct locations inside the placental tissue. Overall placental BC load positively correlated with maternal BC residential exposure averaged over pregnancy, indicating a dose-response relationship. To fully understand direct impacts of ultrafine PM on placental function and offspring health, further studies are needed that consider the toxicity associated with various PM constituents, as well as the role of metabolic activation in placental tissue.

### Placental and systemic maternal oxidative stress and inflammation

Exposure to fine and ultrafine PM elicits maternal systemic and placental oxidative stress and inflammation through various mechanisms [138]. Oxidative stress is generally defined as an imbalance of pro-oxidants and antioxidants driven by an increase in free radicals, namely, reactive oxygen species (ROS). From a mechanistic standpoint, a refined definition also considers oxidative stress a disruption of redox signaling and control without the characteristic involvement of free radicals [137, 139]. PM_2.5_ and PM_0.1_ can induce typical free radical-driven macromolecular damage (i.e., protein modifications, DNA oxidation, and lipid peroxidation), as well as alter the redox states of thiol systems controlled by the thioredoxins (Trx), glutathione (GSH), and cysteine (Cys). The mechanisms of PM_2.5_-associated adverse perinatal outcomes have been previously reviewed [138, 140]. In this section, an update on the mechanisms and evidence in human and animal studies will be discussed.

Briefly, direct generation of ROS from transition metals adsorbed to fine and ultrafine PM, like iron, copper, chromium, and vanadium, work through interactions with superoxide or hydrogen peroxide to form reactive hydroxyl radicals that can damage macromolecules. Metals and organic constituents, like PAHs, adsorbed to PM_2.5_ and PM_0.1_ have high oxidative potential and ability to generate mitochondrial damage [141, 142]. Mitochondrial damage can lead to excess NADPH-oxidase and superoxide production/release. PAHs can also be activated to reactive metabolites via cytochrome (CYP) P450 metabolism. CYPs are transcriptionally upregulated through ligand-activation of the aryl hydrocarbon receptor (AhR). CYP1A1 is the most important xenobiotic-metabolizing enzyme expressed in the placenta, for which activation has been associated with numerous pregnancy-related complications [143]. Toxicological consequences of reactive metabolites, i.e., quinones and epoxides, act through formation of DNA and protein adducts leading to mutagenicity and toxicity. Phase II enzymes, such as glutathione s-transferases (GSTs), UDP-glucuronosyltransferases (UGTs), and NAD(P)H-dependent quinone oxidoreductase-1 (NQO1) detoxify reactive metabolites by catalyzing conjugation reactions and the reduction of quinones to hydroquinones, respectively. Phase II enzymes are transcriptionally upregulated through nuclear factor erythroid 2-related factor (Nrf2) signaling.

Nrf2 is a redox sensitive transcription factor regarded as the master regulator of the antioxidant response [144]. Nrf2 binding to the antioxidant response element (ARE) in promoters upstream of phase II enzymes and other oxidative stress-related enzymes, such as superoxide dismutase (SOD), glutathione peroxidase (GPx), glutathione reductase (GR), and catalase (CAT), drives transcription in response to oxidative stress. The Nrf2 antioxidant response pathway plays an important role in response to PM-induced oxidative stress. Findings from a birth cohort in Korea demonstrating increased susceptibility of lower respiratory tract infections in infants prenatally exposed to PM_2.5_ was significantly modified by polymorphisms in the maternal Nrf2 gene [145]. The risk was increased when investigators included prenatal exposure to environmental tobacco smoke. Additionally, disruption of Nrf2 has been shown to enhance susceptibility to allergic airway inflammatory responses induced by chronic exposure to diesel exhaust PM [146]. Supplementation with thiol antioxidants that activate Nrf2 has been shown to reduce the allergic inflammatory effects of DEPM in vivo [147], a possible preventive intervention approach discussed in the following section. Crosstalk between the AhR and Nrf2 pathways may also be important in response to PM. Shin et al. [148] demonstrated Nrf2 is activated by AhR and can also induce AhR expression. Alternatively, PM-mediated oxidative stress can induce inflammation, sometimes referred to as the tier 2 response preceding tier 3 cytotoxicity or cell death [149, 150]. In the second tier of oxidative stress, the activation of signaling cascades, like the redox-sensitive transcription factor NF-κB, leads to activation of pro-inflammatory cytokines and other immune response genes [151]. Activation of this pathway is associated with increased levels of the pro-inflammatory enzyme COX-2 and cytokines like IL-1β and TNF-α, drivers of systemic inflammation. Complex molecular mechanisms link the Nrf2 and NF-κB pathways. Lack of Nrf2 activity can exacerbate NF-κB signaling, leading to increased cytokine production; alternatively, NF-κB can modulate Nrf2 activity, having both positive and negative effects on target gene expression [152, 153].

Fine PM exposure induces maternal systemic and placental oxidative stress and inflammation, as evidenced in exposed human populations and in experimental models. Nagiah et al. [154] observed pregnant women living in highly industrialized areas of south Durban, South Africa, had increased markers reflective of oxidative stress in circulating lymphocytes as compared to women living in a less industrialized area in the north with lower pollutant levels. Levels of malondialdehyde (MDA), superoxide dismutase (SOD2), and uncoupling protein 2 (UCP2) mRNA were elevated, whereas Nrf2 and GSH expression was decreased in the higher exposure group. Similarly, Ambroz et al. [155] compared urine and blood markers reflecting oxidative DNA damage and lipid peroxidation in mothers and infants living in the Czech Republic in areas with relatively poor to good air quality. Investigators measured 8-oxo-7,8-dihydro-2-deoxyguanosine (8-oxodG), a common product of DNA oxidation, and 15-F2t-isoprostane (15-F2t-IsoP), an oxidized product of arachidonic acid. Isoprostanes, including 15-F2t-IsoP, as well as 8-iso-prostaglandin F2α (8-iso-PGF2α), are routinely employed as markers of oxidative stress. In newborns from the Czech Republic, PM_2.5_ concentrations significantly predicted 8-oxodG excretion, and PM_2.5_ and benzo[a]pyrene concentrations significantly predicted 15-F2t-IsoP levels. In mothers, PM_2.5_ concentrations were a significant predictor of 8-oxodG levels. Using a metabolomics approach, Yan et al. [132] observed maternal oxidative stress and inflammation-related pathways, including linoleate, leukotriene, and prostaglandin, were altered in response to traffic-related air pollution exposure.

Epidemiological studies also reveal positive associations between PM_2.5_ exposure and plasma homocysteine levels, an established marker of cardiovascular disease risk [156]. Homocysteine, a thiol-containing amino acid, is produced by the intracellular demethylation of methionine. Normally, levels of homocysteine are low due to rapid metabolism. In a cohort study, investigators confirmed significantly elevated umbilical cord blood homocysteine levels that were on average 8.1% higher for every 5 μg/m^3^ increase in PM_2.5_ [157]. Interactions with single nucleotide polymorphisms (SNPs) in certain oxidative stress-related enzymes suggested that oxidative stress may be an underlying mechanism. Earlier findings from this cohort revealed placental nitrosative stress, measured via 3-nitrotyrosine concentrations, were significantly associated with increased PM_2.5_ and BC exposure during pregnancy [158]. Markers of mitochondrial damage are also documented in mothers and newborns exposed to PM_2.5_ during gestation. Alterations in mitochondrial DNA (mtDNA) can serve as a marker of cumulative oxidative stress based on the mitochondria’s lack of repair systems. Rosa et al. [159] observed mtDNA content from umbilical cord leukocytes was inversely correlated with PM_2.5_ exposure during pregnancy. These findings were confirmed by Brunst et al. [160], wherein PM_2.5_ exposure across pregnancy was associated with decreased mtDNA copy number in cord blood. Grevendonk et al. [161] demonstrated PM_2.5_ exposure during pregnancy was positively associated with markers of mitochondrial DNA damage in maternal blood.

Telomere length is another measure affected by oxidative stress, and shortening has been related to biological aging. Cord blood and placental telomere length have both been significantly inversely associated with PM_2.5_ exposure [162]. Cord blood leukocyte telomeres were on average 8.8% shorter and placental telomere lengths were 13.2% shorter for every 5 μg/m^3^ increase in PM_2.5_. Rosa et al. [163] reported shorter (but sometimes longer) leukocyte telomere length in association with PM_2.5_ during specific prenatal windows and differences based on neonatal sex. PM_2.5_ was more strongly associated with shortened telomere length in girls as compared to boys. Complex sex and other psycho-social stressor interactions may be important modifiers of markers of oxidative stress, like telomere length and mtDNA. For instance, findings by Brunst et al. [160] indicated the impact of maternal trauma on increased placental mtDNA copy number. In general, psychosocial stress, diet/nutrition, and other maternal exposures like smoking share similar pathways with that of particulate air pollution exposure, which could potentially exacerbate the negative effects of either insult alone [138]. Abundant evidence also indicates gestational PM_2.5_ exposure promotes a systemic pro-inflammatory response and stimulates placental inflammation [164]. Investigators observed PM_2.5_ exposure was associated with increased C-reactive protein (CRP) concentrations in early pregnancy [165]. CRP is a biomarker of systemic inflammation and is often elevated in pregnancy-related conditions [166]. Nachman et al. [167] reported a positive relationship between PM_2.5_ exposure during preconception and pregnancy and intrauterine inflammation. Models of gestational fine and ultrafine PM exposure confirm intrauterine oxidative damage and inflammation [83, 92].

Additionally, there is substantial evidence from animal models to support findings in exposed pregnant women and developing fetuses. Generally, the two mechanisms (PM-induced oxidative stress and inflammation) are studied concurrently. Pregnant mice exposed to DE displayed increased placental expression of immunomodulatory cytokines, including IL-2, IL-5, IL-12, and granulocyte monocyte colony stimulating factor, GM-CSF [102]. In another mouse model, DE exposure increased placental expression of multiple inflammatory cytokines, especially IL-5 and IL-6 (<tenfold) [168]. IL-4 and IL-6 levels have also been shown to increase in response to gestational PM_2.5_ exposure [125]. de Melo et al. [169] further reported increased IL-4 expression in the fetal portion of the placenta in rats exposed to fine PM before and during pregnancy. While most literature agrees that PM_2.5_ induces oxidative stress and inflammatory states, there are a few conflicting results. Biomarkers of systemic inflammation measured in peripheral blood in an ICR mouse model employing gestational PM_2.5_ exposure indicated increased IL-2, IL-6, IL-8, and TNFα [170]. Interestingly, there were no differences in glutathione; however, catalase decreased while heme oxygenase increased indicating PM_2.5_-induced oxidative stress. Pregnant rats exposed to a low dose of PM_2.5_ via intratracheal instillation on gestational days 10 and 18 showed increased IL-6 levels in maternal blood; however, oxidative stress appeared to be less important in this model [89]. There were no differences in GSH-Px and MDA of placenta homogenate between exposed and non-exposed groups. In another mouse model, polluted ambient air exposure from a busy street was associated with thinner umbilical cord walls and increased lipid peroxidation leading to decreased fetal weights [171]. Specifically, the structural changes in umbilical vessels were associated with greater volumes of regions immunostained for 15-F2t-IsoP. Wang et al. [104] confirmed increased 8-isoprostanes in plasma of PM_0.1_-exposed dams. Morales-Rubio et al. [92] verified gestational UFP exposure increased PAH-biotransforming enzymes, intrauterine inflammation and oxidative damage, displayed by increased 8-OHdG in mouse placentae. Last, Song et al. [172] revealed changes in molecular clock gene expression in pregnant rats and their offspring exposed to heavily polluted air. Investigators suggested changes in the regulation of circadian rhythms may be another important pathway for explaining the feedbacks of air pollution exposure in addition to oxidative stress and inflammation. Overall, there is considerable evidence from human and nonhuman studies supporting exposure to fine and ultrafine PM causes adverse effects via oxidative stress/redox signaling imbalance and pro-inflammatory responses.

### Epigenetic alterations

While epigenetic changes in response to fine PM have been documented across lifespan [173], this section will focus on epigenetic alterations following developmental PM_2.5_ exposure. Epigenetics, defined as mitotically or meiotically heritable changes in gene expression without a change in DNA sequence, was first defined by Conrad Waddington in the 1940s [174–176]. Emerging evidence from multiple human cohort studies suggests that gestational fine PM exposure induces several interconnected pathological processes through certain chemical modifications to DNA, mainly methylation, and non-coding RNAs, forming a complex regulatory network that modulates gene expression, contributing to systemic oxidative stress and altered molecular cell signaling pathways. The exact mechanisms regarding PM-induced epigenetic alterations and resulting impact on offspring have been vague and awaits further breakthroughs on many fronts.

DNA methylation is one of the most widely investigated epigenetic modifications and is recognized as one of the mechanisms linking prenatal PM_2.5_ exposure and adverse offspring health outcomes [177]. Studies support gestational PM_2.5_ exposure results in altered DNA methylation, including global hypomethylation in placental tissue [178], as well as gene specific alterations in methylation. It is recognized that epigenetic stability is proportional related to DNA methylation, as global hypomethylation of DNA may lead to genomic instability [179, 180]. Moreover, hypermethylation is associated with developmental defects, such as gestational diabetes and Down’s syndrome [181, 182]. Kingsley et al. [183] also reported lower infant birth weight in association with maternal residence close to a major roadway was correlated with lower mean placental LINE-1 methylation. Moreover, seven CpG sites were significantly associated with residential proximity to major roadways. In a cohort study, exposure to PM_2.5_ during the first trimester was significantly associated with decreased (2.2%) global DNA methylation in placenta tissue [178] and was also significantly correlated with gene expression of S-adenosylmethionine (SAM), a key substrate involved in methyl group transfers [184]. Additionally, investigators reported significant placental DNA methylation in the promoter region of the leptin gene, an energy-regulating hormone involved in fetal growth and development, in association with maternal PM_2.5_ exposure [185]. Zhou et al. [186] linked air pollutant DNA methylation changes directly with oxidative stress, evidenced by significant associations with SOD2 promoter methylation levels in umbilical cord blood. In another cohort, Breton et al. [187] observed prenatal PM_2.5_ exposure was associated with altered DNA methylation in newborn blood in several gene promoters, some which were associated with cardio-respiratory health outcomes later in childhood. Additionally, maternal PM_2.5_ exposure has been correlated with overall placental DNA mutation rate, evidenced by an increased *Alu* rate (as an estimate of global methylation [188]) and altered DNA methylation in the promoter regions of the key DNA repair and tumor suppressor genes including *APEX1*, *ERCC4*, *DAPK*, and *PARP1* [189, 190]. These findings suggest changes to fetal and neonatal DNA repair capacity may play a role in cancer risk later in life. Last, epigenetic modifications in placental mtDNA have been correlated with PM_2.5_ exposure. Epigenetic modifications in mtDNA have been associated with mitochondrial damage and dysfunction and are recognized as an etiological determinant in a variety of diseases, including diabetes, obesity, cardiovascular disease, and cancer [191]. Janssen et al. [192] reported in utero exposure to PM_2.5_ led to lower mtDNA content (as described in the section on oxidative stress), as well as modified the methylation level in the MT-RNR1 region.

Non-coding RNAs, which are not translated into protein, include microRNA (miRNAs), piwi-interacting RNA (piRNA), and long noncoding RNAs (lncRNAs), have been found to participate in various biological processes through interacting with transcriptional factors, repress complex and key regulatory proteins. MicroRNAs, which are endogenous single-stranded small non-coding RNAs of about 22 nucleotides, have been extensively investigated during the past decades and are found to play important regulatory roles in human diseases [193–195]. MiRNAs play a pivotal role in maintaining the healthy condition and regulating the redox state in lungs. Tsamou et al. [196] showed expression of miR-21, miR-146a, and miR-222 significantly decreased in the placental tissues following PM_2.5_ exposure during the second trimester of pregnancy, whereas expression of miR-20a and miR-21 increased in response to fine PM during the first trimester. Mir-21, which is an important regulator in vascular cell proliferation and apoptosis, was also significantly increased in response to diesel exhaust particle and metal-rich PM exposure in adults [197, 198]. Overall, additional research is needed to establish how these early miRNA and mRNA expression changes potentially influence health effects from both fine, as well as ultrafine, PM later in life.

### Endocrine disruption

Evidence on the association between prenatal PM exposure and disorders of the endocrine system and hypothalamic-pituitary-adrenal (HPA) axis are only beginning to emerge. Janssen et al. reported maternal third-trimester exposure to PM_2.5_ was associated with differences in fetal thyroid hormone levels that may contribute to reduced birth weight and long-term health consequences for children [199]. Other mechanisms may involve PM effects on the HPA axis that alter the release of stress hormones like cortisol from the adrenal gland [200]. In a maternal cohort, Khamirchi et al. observed a significant positive association between gestational exposure to PM_2.5_ and cortisol levels in cord blood. The association for PM_1_ exposure was not statistically significant [201]. These results necessitate additional research to confirm findings and evaluate the potential mechanistic linkages between fine and ultrafine fine PM and endocrine-related effects. Moreover, refined measurement of potential endocrine disrupting compounds adsorbed to airborne particles [202] may shed additional light on heterogeneity between studies when only assessing particles based on size.

## Preventive strategies

### Green space to improve air quality

The impact of green space on air pollution exposure and mitigation of health effects in communities has been increasingly studied [203, 204]. A recent report that reviewed the literature on green space, heat, and air pollution generally found that urban green spaces, including trees, parks, green roofs, and large natural space, provide significant health benefits for residents and improvements in air quality [205]. Investigators have also shown children living in areas with more trees have a lower prevalence of early childhood asthma [206]. Specific to prenatal exposure, Dadvand et al. [207] demonstrated lower levels of personal exposure to PM_2.5_ and nitric oxides among pregnant women residing in greener areas in Barcelona, Spain. While few studies have interrogated the potential for green space to reduce maternal and child health effects from fine and ultrafine PM, expansion of green space and access for pregnant women may help combat the adverse health outcomes in children. This is particularly important when looking through the lens of racial inequity in the USA, as studies show historically redlined neighborhoods are still associated with reduced present-day green space [208]. Moreover, racial minority and low socioeconomic status groups experience a disproportionate exposure to air pollution. Across the USA, Blacks are estimated to have a 1.54 times higher burden from PM-emitting facilities than the overall population [209]. Remarkably, the burden for Black Americans was estimated to be higher than the all races living in poverty (1.35 higher burden), indicating disparities based on race may be more pronounced than disparities on the basis of poverty. Thus, combatting racist zoning at the neighborhood and regional levels can help reduce environmental disparities and protect children’s health.

### Nutritional interventions

Nutrition is well recognized as an important modifier along the exposure to disease continuum. Indeed, there is a rich literature related to cancer prevention through the intake of whole foods or simple extracts. The term “green” chemoprevention has been applied to a food-centered approach that may be sustainable in underserved populations [210]. Dietary activation of antioxidant signaling pathways has been proposed as a potential strategy to mitigate pollutant-induced oxidative stress-mediated disease pathogenesis, for instance cancer and chronic lung disease [210, 211]. Clinical trials with broccoli sprouts, rich in the chemoprotective compound glucoraphanin and its biologically active metabolite sulforaphane, in populations highly exposed to air pollution demonstrate the ability of antioxidant pathway activation via Nrf2 to increase metabolism and excretion of airborne pollutants in healthy adults [212]. Since air pollution is an environmental factor that often cannot be avoided, the ability to enhance detoxication of air pollutants and attenuate their associated health risks is of high value, especially in susceptible subgroups.

Studies highlight protective effects from maternal fish consumption, a rich source of omega-3-polusaturated fatty acids (PUFAs). Jedrychowski et al. [213] demonstrated higher maternal exposure to PM_2.5_ was associated with significantly lower infant birth weight; investigators observed greater deficits in infant weight in mothers who reported low fish intake, as compared to medium or high fish consumption. Similarly, in the same cohort, high prenatal PM_2.5_ coupled with postnatal environmental tobacco smoke exposure led to a significantly increased risk for infant eczema, an inflammatory-driven dermatitis [214]. However, when maternal fish intake was high, the risk of infant eczema decreased by 43%, indicating possible benefits of PUFAs for reducing allergic disease. Calderón-Garcidueñas et al. [215] has also reviewed the potential benefit of cocoa flavonols to mitigate PM-associated cognitive and metabolic effects. Flavanols are part of a class of polyphenols known to exert protective effects through their function as antioxidants. In a short-term cocoa intervention, children in the Mexico City Metropolitan Area received 30 grams of dark cocoa with 680 mg of total flavonols; 83% (15/18) of children showed a marginally significant individual improvement in one or both of the applied simple short memory tasks [216]. Moreover, plasma endothelin-1, a potent vasoconstrictor, significantly decreased in response to cocoa intake. It has also been suggested that cocoa and cocoa flavonoids may positively affect endothelial dysfunction and insulin resistance, both known to be associated with PM_2.5_ exposure [217]. Since maternal fine and ultrafine PM exposure has been linked with offspring metabolic disorders via mitochondrial oxidative stress [75], targeting these pathways may also potentially prevent metabolic dysfunction. In an analogous mouse model of maternal tobacco smoke, prenatal exposure led to glucose intolerance, hepatic steatosis, and mitochondrial oxidative stress in adult offspring. Maternal administration of mitoquinone mesylate (MitoQ), a mitochondrial-targeted antioxidant, reduced hepatic mitochondrial oxidative stress and improved markers of mitophagy and mitochondrial biogenesis in offspring [218].

Findings from additional animal models also support the role of antioxidants to mitigate effects in offspring following prenatal PM_2.5_ exposure. Using elegant study design, Repreich et al. [103] demonstrated protection conferred from endotoxin (LPS) on offspring OVA-induced asthma was erased if dams were co-exposed to diesel exhaust PM during pregnancy. This effect was dependent on IFN-γ, and maternal treatment with the antioxidant N-acetyl cysteine (NAC) reversed the IFN-γ-dependent asthma risk in offspring, thereby re-conferring protection. Liu et al. [170] also demonstrated immune protective effects from quercetin, a plant-derived polyphenol, treatment during pregnancy. In this model, gestational PM_2.5_ exposure led to significantly altered T-lymphocyte subsets and increased markers of systemic inflammation and oxidative stress in peripheral blood of pregnant mice. In the quercetin intervention groups, higher doses of supplementation protected the dams against these adverse effects. In an ICR mouse model, gestational exposure to carbon black nanoparticles dose-dependently induced offspring astrogliosis, evidenced by increased reactive astrocytes with glial fibrillary acidic protein (GFAP) and aquaporin-4 overexpression [219]. Maternal antioxidant supplementation with the NAC suppressed GFAP overexpression in offspring, but did not suppress aquaporin-4 overexpression. Interestingly, maternal ascorbic acid (vitamin C) administration did not suppress, but rather slightly enhanced the expression of GFAP and aquaporin-4. These findings emphasize the importance of scrutinizing interventions in vivo, including combined effects, prior to clinical recommendations. This is particularly important in regards to safety of nutrition recommendation and use of dietary supplements during pregnancy. For instance, researchers have reported positive findings in in vitro models showing tea polyphenols ameliorate adverse effects of PM_2.5_ [220]. However, precaution has been advised for the consumption of tea polyphenols and others during late pregnancy based on possible adverse perinatal effects, particularly fetal ductus arteriosus [221].

Folic acid (a B vitamin) is routinely recommended based on evidence that supplementation can prevent neural tube defects [222]. A few studies have investigated the potential for folic acid to mitigate PM-induced health effects in offspring. In a mouse model, B-vitamin supplementation (folic acid, vitamin B6 and vitamin B12) significantly alleviated neurobehavioral impairment in offspring exposed to PM_2.5_ during gestation [223]. Moreover, supplementation increased neurogenesis and reduced synaptic loss and mitochondrial damage in offspring hippocampus. Pro-inflammatory cytokines and oxidative stress-related genes were also downregulated. Since folic acid is an important methyl donor, which is critical to numerous cellular functions, including DNA methylation, the role of folic acid supplementation on DNA methylation changes in the context of PM exposure have been recently interrogated. In a developmental zebrafish model, folic acid was shown to protect against PM_2.5_-induced cardiac toxicity in embryos [224]. Genomic-wide DNA methylation analysis demonstrated both hypo- and hyper-methylation changes in CCGG sites exposed to fine PM that were attenuated by folic acid supplementation [225]. These data suggest folic acid supplementation may protect against developmental cardiac toxicity by mitigating PM_2.5_-induced DNA methylation changes. Further research is required to confirm the protective capacity of folic acid supplementation against PM-induced health effects following developmental exposure.

## Conclusions

In summary, fine and ultrafine PM are important components of ambient air pollution. Developmental exposure is associated with a wide-range of offspring health effects. A large body of observational studies in exposed human populations over decades highlight associations between prenatal fine PM exposure and adverse birth outcomes, namely, low birth weight, respiratory diseases, and adverse neurodevelopment. Recent studies report associations between prenatal fine PM exposure and immune system and metabolic alterations. The most recent State of the Global Air Report [1] for the first time included air pollution-related deaths mediated by low birth weight and preterm birth, which accounted for 20% of neonatal deaths worldwide (1/3 related to PM_2.5_ exposure). Imagine the effect on global morbidity and mortality if all adverse outcomes were to be included.

Additionally, a variety of experimental approaches demonstrate dose-response gradients and support causal associations between developmental fine and ultrafine PM exposure and several adverse outcomes in offspring. Detailed biological mechanisms of action established through molecular epidemiology studies and experimental models support the direct translocation of ultrafine particles across the placental and indirect mechanisms of action, including systemic oxidative stress, inflammation, and epigenetic changes. Additional mechanisms, like how early life air pollution exposure alters the infant microbiome and leads to adverse health outcomes are also beginning to emerge [226] and require increased understanding of how these changes may inform the development of clinical/nutritional strategies.

Overall, improved understanding of the plethora of health impacts stemming from developmental exposure to fine and ultrafine PM supports public health policies that reduce particulate air pollution, particularly for this sensitive population and disproportionally exposed groups. Continued research on health effects of ultrafine PM, source-specific effects, and the effectiveness of mitigation strategies in addition to reducing exposure when policy lags, will aid in reduced health effects in children and overall improved societal outcomes.

## Supplementary Information

**Additional file 1.** Study Selection.

## Data Availability

Data sharing not applicable to this article as no datasets were generated or analyzed during the current study.
