# Supplementary material for: Air pollution and children’s health—a review of adverse effects associated with prenatal exposure from fine to ultrafine particulate matter
Source: Environ Health Prev Med. 2021 Jul 12;26:72. doi: 10.1186/s12199-021-00995-5 (PMC8274666; doi:10.1186/s12199-021-00995-5)
Supplement: Supplementary file 1 — Additional file 1. Study Selection. [file 12199_2021_995_MOESM1_ESM.docx]

**APPENDIX**

**Study Selection**

The result of the PubMed searches (originally conducted January 22, 2020 and updated February 12, 2021 and June 11, 2021).

Prenatal, particulate matter 531 items

Particulate matter, pregnancy, oxidative stress 118 items

Particulate matter, pregnancy, inflammation 110 items

Particulate matter, pregnancy, epigenetic 87 items

Prenatal, particulate matter, endocrine 79 items
